# Time Series Correlations and Kolmogorov Complexity: A Hausdorff Dimension Perspective

**DOI:** 10.3390/e28070812

**Published:** 2026-07-16

**Authors:** Boumediene Hamzi, Marianne Clausel, Kamal Dingle, Marcus Hutter, Mohammed Terry Jack

**Affiliations:** 1 Department of Computing and Mathematical Sciences, California Institute of Technology, Pasadena, CA 91125, USA; 2 The Alan Turing Institute, London NW1 2DB, UK; 3 Institut Élie Cartan de Lorraine, French National Center for Scientific Research (CNRS), Université de Lorraine, 54506 Vandœuvre-lès-Nancy, France; marianne.clausel@univ-lorraine.fr; 4 Department of Mathematics and Natural Sciences, Center for Applied Mathematics and Bioinformatics, Gulf University for Science and Technology, Hawally 32093, Kuwait; dingle.k@gust.edu.kw; 5 School of Computing, Australian National University, Canberra, ACT 2601, Australia; 6 Department of Computer Science, University of York, York YO10 5GH, UK

**Keywords:** spurious correlations, Kolmogorov complexity, algorithmic information theory, Lempel–Ziv complexity, time series, Hausdorff dimension, simplicity bias, fractional Brownian motion

## Abstract

Spurious correlations between time series are a persistent problem: simple, low-complexity patterns are abundant, so unrelated series can easily exhibit high Pearson correlation. We argue that Kolmogorov complexity—a series’ resistance to compression—provides a principled diagnostic for flagging such cases. We prove an algorithmic trilemma: a pair of binary sequences cannot simultaneously be algorithmically independent, highly correlated, and highly complex. This gives a deterministic complexity ceiling for independent correlated pairs and a probabilistic bound under which spurious correlations among independent high-complexity pairs are exponentially rare; we further bridge these results to an effective Hausdorff dimension obstruction. These guarantees hold for binary sequences under Hamming correlation; their extension to real-valued series via serialisation and LZ compression is empirically validated rather than proved, so the joint indicator $J_{\mathrm{LZ}}=\min\left\{{\tilde{C}}_{\mathrm{LZ}}\left(x\right),{\tilde{C}}_{\mathrm{LZ}}\left(y\right)\right\}$ is a calibrated diagnostic, not a causal test. On two toy models—coupled logistic maps and multivariate fractional Brownian motion ($\dim_{H}=2-H$)—false positives are far more common among low-complexity series. Because noise inflates complexity and non-stationary processes can be both complex and spuriously correlated, we recommend a two-stage workflow: establish stationarity, then report $J_{\mathrm{LZ}}$ alongside ρ.

## 1. Introduction

Understanding and predicting time series is fundamental to many areas of mathematics, science, finance, and beyond [1]. Detecting correlations in time series is important across science and engineering [2,3], while distinguishing genuine causal links from coincidental alignment is notoriously difficult [4,5]. Yule [6] identified this problem a century ago, asking “Why do we sometimes get nonsense-correlations between Time-Series?”, and the question remains practically relevant.

**Figure 1:**
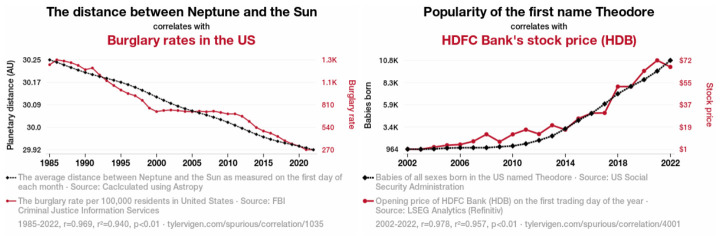
Canonical spurious correlations. Both arise because the underlying series are simple monotone trends with low Kolmogorov complexity. Figures by Tyler Vigen (http://www.tylervigen.com/spurious-correlations (accessed on 1 March 2026)).

The core difficulty is that simple: low-dimensional patterns are abundant in many natural datasets. For example, series taking forms $y\left(t\right)\approx e^{t}$, y(t)≈at+b, or $y\left(t\right)\approx t^{2}$ will appear correlated (at least for short time intervals). More comically, plotting the distance between Neptune and the Sun with burglar rates over a certain time period, or the popularity of the name Theodore and the stock price of HDFC Bank, show how high Pearson correlations can result not because of any fundamental link but because all of these series are themselves simple monotone or near-monotone trends (Figure 1).

Here, we argue that reporting the Kolmogorov complexity of the two series alongside its Pearson correlation provides a principled diagnostic: a high correlation between two high-complexity series constitutes substantially stronger evidence of a genuine relationship than the same correlation between two low-complexity series. For finite binary strings under Hamming correlation, this is more than a heuristic—it follows from a complexity ceiling that we prove in Section 2 and which we extend in Section 2.5.1 to an effective-Hausdorff-dimension obstruction for infinite binary sequences. For real-valued time series, the same principle becomes a *theoretically motivated, empirically calibrated diagnostic*: the binary theorems do not transfer directly through the serialisation–quantisation–compression pipeline, and the operational behaviour of the diagnostic is validated through the toy-model experiments of Section 4 and Section 5 rather than derived from the theorems.

### 1.1. Two Mechanisms, One Workflow

It is useful to distinguish two distinct mechanisms by which spurious correlations arise, because the present paper addresses one and inherits the other from classical econometrics. (i) *Simple deterministic trends*—monotone or near-monotone patterns of low algorithmic complexity, exemplified by the Tyler Vigen catalogue (Figure 1)—produce high |ρ| because both series happen to share an elementary functional form. This is the regime targeted directly by our complexity screen. (ii) *Integrated non-stationary stochastic processes*—random walks, fBm paths, and other unit-root-type series—also produce high |ρ| between independent realisations, the “nonsense correlations” identified by Yule [6] and analysed for econometric regressions by Granger and Newbold [7]. These are *algorithmically complex* (Section 5) and are therefore invisible to a pointwise complexity screen until they are differenced to stationary increments.

We therefore advance the *stationarity-first, complexity-second workflow* as a central methodological contribution rather than as a caveat: the stationarity step (testing for a unit root, differencing, or detrending) targets Yule-type non-stationary stochastic correlations, while the complexity screen (computing $J_{\mathrm{LZ}}$ and comparing against a calibrated threshold or family rule) targets correlations plausibly driven by shared low-complexity deterministic structure. Neither step alone is sufficient: complexity ignores integrated processes, and stationarity testing ignores trend-driven coincidences. The full algorithm is given in Section 7; we make the two-stage structure explicit throughout this paper.

This workflow defines the intended boundary of applicability. In bounded dynamical settings where the serialised complexity proxy has real dynamic range, $J_{\mathrm{LZ}}$ is a cheap marginal-complexity screen, and an operating threshold such as θ=0.3 can be calibrated directly. In stationary Gaussian-increment settings such as fGn under fine serialisation, pointwise LZ complexity saturates, so $J_{\mathrm{LZ}}$ should not be advertised as an absolute or rank-based cutoff; the relevant output is instead the false-positive risk as a function of an estimated memory/smoothness family parameter such as *H*. Thus, estimating *H* is not a competitor to the diagnostic but the correct calibration coordinate for this particular data class, while $J_{\mathrm{LZ}}$ retains its screening role in data classes whose encoding preserves complexity contrast.

### 1.2. Why Existing Diagnostics Leave a Gap

Three classes of existing methods address spurious correlations partially. Permutation and phase-randomisation surrogate tests [8] reject the chosen null hypothesis when the observed correlation is inconsistent with an i.i.d. or linear-Gaussian null, but a pair of simple monotone trends typically passes such tests because the correlation is real *against the chosen null*—it is the null itself that is inappropriate. Cointegration and unit-root tests [9] address mechanism (ii) above but say nothing about mechanism (i). MDL-based causal-discovery methods [10,11] are structurally heavyweight: they aim to recover a full directed acyclic graph and are not designed for the question “is this single Pearson correlation worth trusting?”. The complexity screen we propose fills this lightweight, single-pair gap, complementing rather than replacing the three classes above (see Section 6 for the detailed contrast).

This paper is organised as follows: Section 2 develops the theoretical framework connecting Kolmogorov complexity, Hausdorff dimension, and spurious correlations, including the noise-complexity link. Section 3 describes our complexity estimators and the improved joint indicator. Section 4 presents experiments on coupled logistic maps. Section 5 presents experiments on multivariate fractional Brownian motion. Section 6 compares our approach with existing spurious-correlation diagnostics. Section 7 consolidates threshold selection and sensitivity into a single practical recipe. Section 8 illustrates the framework on stylised real-world patterns. Section 9 discusses implications, limitations, and connections to causal inference.

An important caveat, discussed in detail in Remark 3, is that the proposed framework assumes approximately stationary series. Non-stationarity—such as stochastic trends or structural breaks—can inflate LZ complexity estimates and should be diagnosed and corrected (e.g., by differencing or detrending) before applying our screening indicators.

## 2. Theoretical Framework

This section develops the algorithmic-information-theoretic foundation of this paper. We begin by reviewing Kolmogorov complexity and the Solomonoff prior (Section 2.1 and Section 2.2), which explain why simple patterns are overrepresented in naturally generated data. We then turn to the core theoretical contribution: an algorithmic trilemma stating that a pair of binary sequences cannot simultaneously be independent, highly correlated, and highly complex (Section 2.4). We formalise this as two propositions—a deterministic complexity ceiling for algorithmically independent pairs, and a probabilistic alignment bound for statistically independent pairs drawn from the Solomonoff prior. We connect the complexity rate of individual sequences to the classical Hausdorff dimension of sets (Section 2.5), discuss how measurement noise inflates observed complexity via the Posobin–Shen theorem (Section 2.6), and close with the connection to algorithmic causality (Section 2.7).

### 2.1. Kolmogorov Complexity

Within theoretical computer science, *algorithmic information theory* (AIT) [12,13,14] connects computability theory and information theory. The central quantity of AIT is *Kolmogorov complexity*, K(x), which measures the complexity of an individual object *x* as the amount of information required to describe or generate *x*. K(x) is more technically defined as the length of a shortest program which runs on an optimal prefix *universal Turing machine* (UTM) [15], generates *x*, and halts. Intuitively, K(x) is a measure of the compressed version of a data object. Objects containing simple or repeating patterns like 010101010101 will have low complexity, while objects lacking patterns will have high complexity.

The most commonly used variant of Kolmogorov complexity is *prefix complexity* [16,17], also denoted K(x) (and its conditional version K(x|y)), which is defined as the minimum length of a program which generates some output *x* (given side information *y*) and halts when run on a prefix universal Turing machine *U*:

(1) $$K\left(x\right)=\min_{p}\left\{|p|:U\left(p\right)=x\right\}\;\;\;\mathrm{and}\;\;\;K\left(x|y\right)=\min_{p}\left\{|p|:U\left(p,y\right)=x\right\}$$

K(x) is formally uncomputable, meaning that there cannot exist an algorithm that takes any arbitrary string *x* and returns the complexity value [16]. This uncomputability is related to the Halting Problem and, more broadly, to the logical problems of self-referential statements. Real-world applications of Kolmogorov complexity typically rely on approximations to this uncomputable quantity, and these mostly take the form of data compression algorithms such as methods inspired by Lempel–Ziv (LZ) complexity [18]. The use of LZ compression to approximate Kolmogorov complexity in dynamical systems has been explored in several works, including the computable-information-density framework of Benci, Bonanno, Galatolo, Menconi, and Virgilio [19] and the compression-based time-sequence analysis of Puglisi, Benedetto, Caglioti, Loreto, and Vulpiani [20], as well as applications to entropy estimation in neural and chaotic systems [21,22]. While real-world compression algorithms will fall short of accurately estimating the true Kolmogorov complexity, approximations often work very well at capturing meaningful features of complexity distributions [23,24,25,26,27].

### 2.2. Algorithmic Probability

An important result in AIT is Levin’s *coding theorem* [28], which establishes a fundamental connection between K(x) and probability predictions. Mathematically, it states that

(2) $$\mathrm{Pr}\left[x\right]\approx 2^{-K(x)}$$

where Pr[x] is the probability that a (prefix-optimal) universal Turing machine, when fed with a random binary program, outputs *x*. Probability estimates based on the Kolmogorov complexity of output patterns are called *algorithmic probability*. We will also refer to Equation (2) as the *Solomonoff prior*.

While directly applying algorithmic probability in real-world applications is problematic (due to uncomputability), approximations to algorithmic probability for real-world input–output maps have been developed, leading to the observation of a phenomenon known as *simplicity bias* [27,29]. This shows that even though algorithmic probability is an abstract concept from computer science, it can usefully be applied in real-world settings, including, e.g., statistical data analysis.

### 2.3. Spurious Correlations

According to the Solomonoff prior, strings with low K(x) are exponentially more probable under the universal distribution. This means that simple patterns such as monotone trends, periodic sequences, and simple polynomial functions are much more likely to appear in naturally generated data, as compared to if the data were generated by coin flips. See [30,31] for more discussion and empirical exploration of simplicity bias in natural time series.

It follows that two independently generated simple sequences are far more likely to coincide than two independently generated complex ones (cf. results in [32]), or at least be correlated. The next subsection makes this intuition precise through an algorithmic trilemma: a pair of binary sequences cannot simultaneously be independent, highly correlated, and highly complex.

### 2.4. Incompatibility of Algorithmic Independence, High Complexity, and Spurious Correlations

We formalise a fundamental trilemma in Algorithmic Information Theory concerning pairs of binary sequences: a pair cannot simultaneously exhibit independence, high complexity, and high empirical correlation. We explore this trilemma through two dual frameworks. First, we prove a deterministic pointwise bound demonstrating that algorithmically independent strings cannot exceed a complexity ceiling determined by their correlation. Second, we prove a conditional probabilistic bound showing that statistically independent, highly complex generative processes yield spuriously correlated sequences with exponentially decaying probability.

#### 2.4.1. The Algorithmic Trilemma

Consider two binary sequences of length *n*. We wish to analyse three specific properties that a pair of such sequences might possess:

1. *Independence*: the sequences share no hidden mutual information.
2. *High Correlation*: the sequences are structurally very similar (e.g., a high Pearson correlation coefficient).
3. *High Complexity*: the sequences contain dense algorithmic information (i.e., they are incompressible).

The underlying physical law in Algorithmic Information Theory governing these properties is a strict trilemma: *You can only pick two.* Table 1 summarises these three pairwise regimes.

If two sequences are highly correlated and highly complex, they must be mathematically dependent, sharing significant mutual information to achieve that complex alignment. If they are independent and complex, they will look like distinct white noise and lack structural correlation. If they are independent and highly correlated, they must both be highly simple (e.g., two strings of all zeros).

This trilemma can be formalised from two different perspectives: deterministic (algorithmic) independence and probabilistic (statistical) independence.

#### 2.4.2. Preliminaries and Definitions

To avoid notational conflict with the real-valued time series $x,y\in R^{N}$ of Section 3, Section 4 and Section 5, we use lightface u,v (and capitals U,V for random variables) for binary strings throughout this section.

Throughout this section, the length satisfies n≥1: the normalised complexity $\tilde{K}\left(u\right)=K\left(u\right)/n$ and the Hamming correlation $\hat{\rho }\left(u,v\right)=1-2d_{H}\left(u,v\right)/n$ defined below are undefined at n=0, and the asymptotic (effective-dimension) statements are understood in the limit n→∞. For $u\in {\left\{0,1\right\}}^{n}$, the normalised complexity is defined as $\tilde{K}\left(u\right)=K\left(u\right)/n\in \left[0,1\right]$. Two strings $u,v\in {\left\{0,1\right\}}^{n}$ are *c*-*algorithmically independent* if knowing *u* does not help compress *v*, formally if K(u,v)≥K(u)+K(v)−c. The *normalised Hamming Correlation* (also called *bipolar overlap*) for $u,v\in {\left\{0,1\right\}}^{n}$ is defined as $\hat{\rho }\left(u,v\right)=1-2d_{H}\left(u,v\right)/n$, where $d_{H}$ is the Hamming distance. Thus, a correlation $\hat{\rho }\left(u,v\right)>\tau >0$ confines *v* to a Hamming ball around *u* of radius exactly $\delta_{\tau }n$, where $\delta_{\tau }=\frac{1-\tau }{2}<\frac{1}{2}$. The number of strings in this ball is bounded by $2^{nh(\delta_{\tau })}$, where $h\left(p\right)=-p\log_{2}p-\left(1-p\right)\log_{2}\left(1-p\right)$ is the binary entropy function. The *length-conditioned Solomonoff-type prior* for $u\in {\left\{0,1\right\}}^{n}$ is defined as $M_{n}\left(u\right):=2^{-K(u)}/Z_{n}$, where the normalisation constant $Z_{n}:=\sum_{z\in {\left\{0,1\right\}}^{n}}2^{-K(z)}$. This is a length-conditioned normalisation of the universal a priori weights $2^{-K(u)}$ over strings of fixed length *n*; it should not be confused with the unconditioned universal semimeasure.

#### 2.4.3. Pearson vs. Hamming Correlation

$\hat{\rho }\left(u,u\right)=1$ and $\hat{\rho }\left(u,\bar{u}\right)=-1$, where $\bar{u}$ is the bitwise complement. The same is true for the empirical Pearson correlation coefficient (see Definition A1 in Appendix A or Equation (3)). Indeed, they closely match each other in general but are not exactly equal (if *u* and *v* are each balanced, then both correlations coincide). While the propositions below are formulated using this Hamming correlation for geometric clarity, essentially the same capacity bounds hold for the standard empirical *Pearson correlation*. Bounding the Pearson volume requires an empirical entropy bound rather than a simple Hamming ball, resulting in an additional $O(n^{2})$ polynomial factor which in Proposition 1 is absorbed in the O() term and in Proposition 2 increases the $O(n^{2})$ prefactor to $O(n^{4})$. The proof of this Pearson volume bound is provided in Appendix A.

#### 2.4.4. The Duality of Independence

The trilemma can be expressed through two distinct frameworks of independence:

*Algorithmic Independence* focuses on the final, realised sequences. It states that if you assume strings are both algorithmically independent and highly correlated, they are mathematically forbidden from crossing a specific “complexity ceiling”.

*Statistical Independence* focuses on the generative processes. It models two isolated rooms flipping coins governed by the universal prior $M_{n}$. The physical processes are independent, so their joint probability factorises. In this framework, we can enforce the condition that the sequences *must* be highly complex. The trilemma dictates that any highly correlated, highly complex pair of strings must be algorithmically dependent. For statistically independent processes to generate such an algorithmically dependent pair, they must do so by pure, astronomically rare luck.

We first prove that algorithmic independence and high correlation upper-bound the allowable complexity of the individual strings.

**Proposition 1** 
(Deterministic complexity ceiling)**.** *Let $u,v\in {\left\{0,1\right\}}^{n}$ be c-algorithmically independent. If u and v exhibit an empirical correlation $\hat{\rho }\left(u,v\right)>\tau >0$, then*

$$\max\left\{K\left(u\right),K\left(v\right)\right\}\;\leq \;n\;h\left(\delta_{\tau }\right)+c+O\left(\log n\right),$$

*or equivalently, after dividing by n, both of their normalised Kolmogorov complexities are bounded away from 1:*

$$\max\left\{\tilde{K}\left(u\right),\tilde{K}\left(v\right)\right\}\;\leq \;h\left(\delta_{\tau }\right)+\frac{c}{n}+O\;\left(\frac{\log n}{n}\right)\;\;\;\mathrm{where}\;\;\;\delta_{\tau }=\frac{1-\tau }{2}.$$

*The bound is asymptotically informative only when c=o(n); the algorithmic-independence constant c contributes additively to the complexity ceiling at the unnormalised level and as c/n at the normalised level. Throughout this paper, we work in the regime c=o(n).*

**Proof.**  Assume $\hat{\rho }\left(u,v\right)>\tau$. As established geometrically, *v* must reside in a restricted Hamming ball surrounding *u* of cardinality at most $2^{nh(\delta_{\tau })}$.

An algorithm can perfectly reproduce *v* if provided with *u* and the specific index of *v* within that bounded ball. Storing this index requires at most $\log_{2}\left(2^{nh(\delta_{\tau })}\right)=nh\left(\delta_{\tau }\right)$ bits. Therefore, the conditional Kolmogorov complexity is bounded:

$$K\left(v|u\right)\;\leq \;nh\left(\delta_{\tau }\right)+O\left(\log n\right)$$

The O(logn) takes care of encoding τ to sufficient accuracy. By the chain rule of Kolmogorov complexity, the joint complexity is K(u,v)=K(u)+K(v|u)+O(logn). Substituting this into the *c*-algorithmic independence assumption K(u)+K(v)−c≤K(u,v), we obtain:

$$K\left(u\right)+K\left(v\right)-c\;\leq \;K\left(u\right)+nh\left(\delta_{\tau }\right)+O\left(\log n\right)$$

Subtracting K(u) from both sides yields:

$$K\left(v\right)\;\leq \;nh\left(\delta_{\tau }\right)+O\left(\log n\right)+c$$

By symmetry, swapping the roles of *u* and *v* yields the same bound on K(u). Dividing by *n* completes the proof. Because $\tau >0\Rightarrow h(\delta_{\tau })<1$, neither independent sequence is permitted to be uniformly complex.    □

We now shift to the probabilistic framework. We ask the following: given that two statistically independent processes generate highly complex sequences, what is the probability they randomly exhibit a spurious correlation?

**Proposition 2** 
(Spurious probabilistic alignment bound)**.** *Let $U,V\in {\left\{0,1\right\}}^{n}$ be independent random variables drawn from the length-conditioned Solomonoff-type prior $M_{n}$. For any correlation threshold τ>0 and complexity thresholds α,β∈(0,1), provided $\max\left\{\alpha ,\beta \right\}>h\left(\delta_{\tau }\right)$, the conditional probability of a spurious high correlation given that both strings are complex is exponentially small:*

$$\underset{U,V∼M_{n}}{\mathrm{Pr}}\left(\hat{\rho }\left(U,V\right)>\tau \;|\;\tilde{K}\left(U\right)\geq \alpha \;\mathrm{and}\;\tilde{K}\left(V\right)\geq \beta \right)\;\leq \;O\left(n^{2}\right)\cdot 2^{-n\left(\max\left\{\alpha ,\beta \right\}-h\left(\delta_{\tau }\right)\right)}$$

We need $\alpha \vee \beta >h(\delta_{\tau })$ to achieve exponential decay, where $\delta_{\tau }=\left(1-\tau \right)/2$. For instance, for τ=0.7, we have $\delta_{\tau }=0.15$ and $h(\delta_{\tau })\approx 0.61$, so a complexity threshold α=0.7 lies above the entropy ceiling by roughly 0.09; the decay is then about $2^{-0.09n}$, consistent with Proposition 1 implying that $\max\left\{\tilde{K}\left(u\right),\tilde{K}\left(v\right)\right\}≲h\left(\delta_{\tau }\right)\approx 0.61$ for sufficiently long algorithmically independent strings.

**Proof.**  Let $C_{U}$ and $C_{V}$ denote the respective events that the strings are complex: $\tilde{K}\left(U\right)\geq \alpha$ and $\tilde{K}\left(V\right)\geq \beta$. By the definition of conditional probability for statistically independent variables:

$$\mathrm{Pr}\left(\hat{\rho }>\tau |C_{U}\cap C_{V}\right)\;=\;\frac{\mathrm{Pr}(\hat{\rho }\left(U,V\right)>\tau \;\wedge \;U\in C_{U}\;\wedge \;V\in C_{V})}{\mathrm{Pr}\left(U\in C_{U}\right)\mathrm{Pr}\left(V\in C_{V}\right)}$$

Let $S_{\tau }\left(u\right)=\left\{v\in {\left\{0,1\right\}}^{n}:\hat{\rho }\left(u,v\right)>\tau \right\}$ be the Hamming ball of highly correlated matches around *u*, where $|S_{\tau }\left(u\right)|\leq 2^{nh(\delta_{\tau })}$.

*Step 1: Bounding the Numerator.* We expand the joint probability in the numerator by marginalising over *u*:

$$\mathrm{Numerator}\;=\sum_{u\in C_{U}}M_{n}\left(u\right)\left(\sum_{v\in C_{V}\cap S_{\tau }\left(u\right)}M_{n}\left(v\right)\right)$$

For every *v* in the inner sum, $v\in C_{V}$, implying K(v)≥βn. Its weight is bounded by $M_{n}\left(v\right)\leq \frac{2^{-\beta n}}{Z_{n}}$. Bounding the inner sum by its maximum volume times the maximum uniform weight gives:

$$\sum_{v\in C_{V}\cap S_{\tau }\left(u\right)}M_{n}\left(v\right)\;\leq \;\left|S_{\tau }\left(u\right)\right|\cdot \frac{2^{-\beta n}}{Z_{n}}\;\leq \;\frac{2^{-n(\beta -h\left(\delta_{\tau }\right))}}{Z_{n}}$$

Substituting this bound back into the outer sum over *u* reveals:

$$\mathrm{Numerator}\;\leq \;\left(\sum_{u\in C_{U}}M_{n}\left(u\right)\right)\frac{2^{-n(\beta -h\left(\delta_{\tau }\right))}}{Z_{n}}\;\;=\;\;\mathrm{Pr}\left(U\in C_{U}\right)\cdot \frac{2^{-n(\beta -h\left(\delta_{\tau }\right))}}{Z_{n}}$$

*Step 2: Algebraic Cancellation.* Substituting the bounded numerator into the Bayes fraction causes $\mathrm{Pr}(U\in C_{U})$ to cancel:

$$\mathrm{Pr}\left(\hat{\rho }>\tau |C_{U}\cap C_{V}\right)\;\leq \;\frac{\mathrm{Pr}\left(U\in C_{U}\right)\cdot \frac{2^{-n(\beta -h\left(\delta_{\tau }\right))}}{Z_{n}}}{\mathrm{Pr}\left(U\in C_{U}\right)\mathrm{Pr}\left(V\in C_{V}\right)}\;\;=\;\;\frac{2^{-n(\beta -h\left(\delta_{\tau }\right))}}{Z_{n}\mathrm{Pr}\left(V\in C_{V}\right)}$$

This cancellation demonstrates that conditioning on the complexity of *U* mathematically shields us from paying the probability penalty of generating it.

*Step 3: Lower-Bounding the Denominator Mass.* We must ensure the remaining denominator $Z_{n}\mathrm{Pr}\left(V\in C_{V}\right)=\sum_{v\in C_{V}}2^{-K(v)}$ does not decay exponentially, which would weaken the bound. Out of $2^{n}$ strings, at most $\sum_{k=0}^{n-2}2^{k}=2^{n-1}-1$ have complexity strictly less than n−1. Thus, there are more than $2^{n-1}$ sequences with K(v)≥n−1. For sufficiently large *n*, n−1≥βn, ensuring these sequences belong to $C_{V}$.

By the Invariance Theorem, a universal Turing machine can output any string by reading its length (≤$2\log_{2}n+O\left(1\right)$ bits) followed by its literal bits. Thus, uniformly, $K\left(v\right)\leq n+2\log_{2}n+O\left(1\right)$. Summing this minimum weight over the volume of complex strings guarantees a polynomial lower bound:

$$\sum_{v\in C_{V}}2^{-K(v)}\;\geq \;2^{n-1}\cdot 2^{-n-2\log_{2}n-O\left(1\right)}\;\geq \;\Omega \left(\frac{1}{n^{2}}\right)$$

*Step 4: Final Synthesis.* Substituting this polynomial lower bound into Step 2 yields:

$$\mathrm{Pr}\left(\hat{\rho }>\tau |C_{U}\cap C_{V}\right)\;\leq \;\frac{2^{-n(\beta -h\left(\delta_{\tau }\right))}}{\Omega (n^{-2})}\;\leq \;O\left(n^{2}\right)\cdot 2^{-n(\beta -h\left(\delta_{\tau }\right))}$$

By symmetric marginalisation (evaluating over *v* first to cancel $\mathrm{Pr}(V\in C_{V})$), we obtain the independent constraint $O\left(n^{2}\right)\cdot 2^{-n(\alpha -h\left(\delta_{\tau }\right))}$. Because the true conditional probability must simultaneously satisfy both upper bounds, we take the minimum of the two limits, yielding the final exponent:

$$\mathrm{Pr}\left(\hat{\rho }>\tau |C_{U}\cap C_{V}\right)\;\leq \;O\left(n^{2}\right)\cdot 2^{-n\left(\max\left\{\alpha ,\beta \right\}-h\left(\delta_{\tau }\right)\right)}$$

   □

The algorithmic bound (Proposition 1) dictates that crossing the complexity ceiling is logically impossible for *algorithmically* independent strings. The probabilistic bound (Proposition 2) measures the exponentially tiny probability of *statistical* independent processes accidentally generating dependent strings that violate this ceiling. Together, they formalise the boundaries of spurious structural alignment.

#### 2.4.5. From Theory to Practice

Propositions 1 and 2 provide rigorous bounds for *binary* strings of length *n* under Hamming correlation (with Pearson correlation handled by the polynomial-factor reduction noted above and proved in Appendix A); their asymptotic counterpart is the effective-Hausdorff-dimension obstruction stated in Section 2.5.1 (Propositions 3 and 4). The practical pipeline introduced in Section 3 extends this to real-valued time series through two additional approximation steps: (i) serialisation of floating-point values to byte strings (Section 3.2.1), and (ii) the use of LZ compression as a computationally tractable proxy for the uncomputable Kolmogorov complexity. Both steps are well motivated—any lossless computable compression scheme yields an upper bound on K(x) [16]—but neither is covered by the formal guarantees of the propositions. Section 3.1 makes this layering explicit through a four-level taxonomy (binary strings → real vectors → serialised byte strings → compressed byte strings) and emphasises that the $J_{\mathrm{LZ}}$ procedure should be interpreted as a theoretically motivated, empirically calibrated diagnostic operating at the deepest level rather than as a direct consequence of the binary theorems. The experimental validation on coupled logistic maps (Section 4) and fractional Brownian motion (Section 5) demonstrates that the qualitative prediction of the trilemma (fewer spurious correlations at higher complexity) holds robustly for real-valued series under compression-based proxies. Closing this gap formally—by extending the bounds to quantised real-valued sequences and bounding the approximation error of LZ compression relative to true Kolmogorov complexity—is an important direction for future work.

#### 2.4.6. Relation to the $J_{\mathrm{LZ}}$ Indicator

Proposition 2 shows that exponential decay of spurious alignment requires $\max\left\{\alpha ,\beta \right\}>h\left(\delta_{\tau }\right)$—i.e., at least one of the two series must be sufficiently complex. The practical screening indicator $J_{\mathrm{LZ}}$ introduced in Section 3 imposes the strictly stronger condition that *both* series be individually complex. Section 3 motivates this choice empirically: on asymmetric pairs, an indicator based on max (closer to the theorem’s minimal hypothesis) is too permissive, flagging pairs as trustworthy when one series is a simple trend.

### 2.5. Hausdorff Dimension and the KC-Dimension Bridge

In mathematics, the Hausdorff dimension provides a rigorous metric for characterising the “roughness” or fractal complexity of a set. Developed by Felix Hausdorff in 1918, it generalises the intuitive notion of dimension by allowing for non-integer values. For standard Euclidean objects, the Hausdorff dimension aligns with topological dimension: A singleton point has a dimension of 0; a line segment has a dimension of 1; a square has a dimension of 2; a cube has a dimension of 3.

For sets defining smooth manifolds or polytopes, typical of classical geometry, the Hausdorff dimension is an integer equivalent to the dimension of the space the shape occupies. For infinite sequences, the connection between Kolmogorov complexity and geometric complexity is made precise by the following foundational result.

**Definition 1** 
(Complexity rate/normalised Kolmogorov complexity)**.** *The complexity rate (or normalised Kolmogorov complexity ) of an infinite sequence $x\in {\left\{0,1\right\}}^{N}$ is*

$$\kappa \left(x\right)\;=\;\underset{r\to \infty }{\lim\inf}\frac{K(x↾r)}{r},$$

*where x↾r denotes the prefix of length r. This quantity measures the asymptotic compressibility of individual sequences: κ(x)=0 for eventually periodic sequences and κ(x)=1 for algorithmically random ones.*

**Definition 2** 
(Kolmogorov complexity relative to an oracle)**.** *For an oracle set A⊆N, the Kolmogorov complexity relative to A is $K^{A}\left(x\right)=\min\{|p|:U^{A}\left(p\right)=x\}$, where $U^{A}$ is a universal prefix-free Turing machine with oracle access to A. The unconditional complexity K(x) corresponds to A=⌀. The complexity rate relative to A is $\kappa^{A}\left(x\right)={\lim\inf}_{r\to \infty }K^{A}\left(x↾r\right)/r$.*

The connection between the complexity rate of individual sequences and the Hausdorff dimension of sets is made precise by the foundational result of Lutz [33]: the complexity rate κ(x) coincides with the *effective Hausdorff dimension* dim(x) of the sequence; the corresponding generalisation of this complexity-rate notion to general metric spaces was given by Galatolo [34]. More generally, the classical Hausdorff dimension of a set can be recovered from the complexity rates of its points via the *point-to-set principle* [35]: for any set $E\subseteq {\left\{0,1\right\}}^{N}$,

$$\dim_{H}\left(E\right)\;=\;\min_{A\subseteq N}\underset{x\in E}{\sup}\kappa^{A}\left(x\right),$$

where the minimum is over all oracle sets *A*. In words, the Hausdorff dimension of a set equals the supremum of the oracle-relative complexity rates of its points, minimised over all possible oracles. Equivalently, for the classical Hausdorff dimension of the graph of a fractional Brownian motion (fBm) path with Hurst parameter *H*,

$$\dim_{H}\left(\mathrm{graph}\;\mathrm{of}\;{\mathrm{fBm}}_{H}\right)=2-H.$$

#### 2.5.1. Effective Hausdorff Dimension Bridge

Proposition 1 is a finite-string statement: it controls $\max\{\tilde{K}\left(u\right),\tilde{K}\left(v\right)\}$ at fixed length *n*. The natural asymptotic counterpart is an obstruction on the *effective Hausdorff dimension* of infinite binary sequences. We state this obstruction in two forms—persistent and subsequential—and discuss its scope.

##### Scope

The theorem below is an asymptotic result about infinite binary sequences under Hamming correlation. It rigorously bridges the finite Hamming-ball complexity ceiling of Proposition 1 to the effective-dimension framework of Lutz [33] and Staiger [36], justifying the Hausdorff-dimension framing of the binary theory. It does *not* apply directly to real-valued time series, Pearson correlation, fractional Brownian motion sample paths, or LZ-compressed serialised data; for those, the empirical pipeline of Section 3, Section 4 and Section 5 and the $J_{\mathrm{LZ}}$-as-diagnostic interpretation apply instead.

For $x\in {\left\{0,1\right\}}^{N}$, write x↾n for the length-*n* prefix. Define the *effective Hausdorff dimension* of *x* as in Definition 1, $\dim_{\mathrm{eff}}\left(x\right):=\kappa \left(x\right)={\lim\inf}_{n\to \infty }K\left(x↾n\right)/n$, and the *upper effective dimension* (or effective packing dimension) by ${\mathrm{Dim}}_{\mathrm{eff}}\left(x\right):={\mathrm{lim sup}}_{n\to \infty }K\left(x↾n\right)/n$.

**Proposition 3** 
(Effective-dimension obstruction to persistent correlation)**.** *Let $x,y\in {\left\{0,1\right\}}^{N}$, and suppose*

I(x↾n:y↾n)=o(n)

*(algorithmic independence up to o(n) mutual information). Fix 0<τ<1 and set $\delta_{\tau }=\left(1-\tau \right)/2$. If for all sufficiently large n,*

$$\hat{\rho }\left(x↾n,\;y↾n\right)\;\geq \;\tau ,$$

*then*

$${\mathrm{Dim}}_{\mathrm{eff}}\left(x\right)\;\leq \;h\left(\delta_{\tau }\right),\;{\mathrm{Dim}}_{\mathrm{eff}}\left(y\right)\;\leq \;h\left(\delta_{\tau }\right),$$

*and consequently $\dim_{\mathrm{eff}}\left(x\right)\leq h\left(\delta_{\tau }\right)$ and $\dim_{\mathrm{eff}}\left(y\right)\leq h\left(\delta_{\tau }\right)$. Equivalently (the exact contrapositive), if $\max\left\{{\mathrm{Dim}}_{\mathrm{eff}}\left(x\right),{\mathrm{Dim}}_{\mathrm{eff}}\left(y\right)\right\}>h\left(\delta_{\tau }\right)$, then x and y cannot be simultaneously algorithmically independent up to o(n) mutual information and τ-correlated for all sufficiently large n. In particular, the same conclusion holds under the stronger condition $\min\left\{{\mathrm{Dim}}_{\mathrm{eff}}\left(x\right),{\mathrm{Dim}}_{\mathrm{eff}}\left(y\right)\right\}>h\left(\delta_{\tau }\right)$, which expresses the intended interpretation that both sequences have high effective dimension.*

**Proof.**  Let $u_{n}=x↾n$, $v_{n}=y↾n$. The correlation assumption gives $\hat{\rho }\left(u_{n},v_{n}\right)\geq \tau$ eventually, i.e., $d_{H}\left(u_{n},v_{n}\right)\leq \delta_{\tau }n$. Hence, $v_{n}$ lies in the Hamming ball $B(u_{n},\delta_{\tau }n)$, whose cardinality is at most $2^{n\;h(\delta_{\tau })}$ by the standard entropy bound. Conditional on $u_{n}$, the string $v_{n}$ can be encoded by its index inside this ball using at most $n\;h(\delta_{\tau })$ bits, plus O(logn) bits for the length *n* and decoding conventions. Therefore

$$K\left(v_{n}|u_{n}\right)\;\leq \;n\;h\left(\delta_{\tau }\right)+O\left(\log n\right).$$

By the chain rule, $K\left(u_{n},v_{n}\right)\leq K\left(u_{n}\right)+K\left(v_{n}|u_{n}\right)+O\left(\log n\right)$. Combining with the o(n)-independence assumption $K\left(u_{n}\right)+K\left(v_{n}\right)\leq K\left(u_{n},v_{n}\right)+o\left(n\right)$ and cancelling $K(u_{n})$ yields

$$K\left(v_{n}\right)\;\leq \;n\;h\left(\delta_{\tau }\right)+o\left(n\right).$$

Dividing by *n* and taking ${\mathrm{lim sup}}_{n\to \infty }$ gives ${\mathrm{Dim}}_{\mathrm{eff}}\left(y\right)\leq h\left(\delta_{\tau }\right)$, hence also $\dim_{\mathrm{eff}}\left(y\right)\leq h\left(\delta_{\tau }\right)$. Interchanging the roles of $u_{n}$ and $v_{n}$ in the conditional-encoding step yields the same bound for *x*.    □

**Proposition 4** 
(Subsequential effective-dimension obstruction)**.** *Under the same independence assumption I(x↾n:y↾n)=o(n), if there exists an infinite sequence $n_{j}\to \infty$ such that $\hat{\rho }\left(x↾n_{j},\;y↾n_{j}\right)\geq \tau$ for every j, then*

$$\dim_{\mathrm{eff}}\left(x\right)\;\leq \;h\left(\delta_{\tau }\right)\;\mathrm{and}\;\dim_{\mathrm{eff}}\left(y\right)\;\leq \;h\left(\delta_{\tau }\right).$$

**Proof.**  Apply the Hamming-ball argument along the subsequence $n_{j}$ to obtain $K\left(y↾n_{j}\right)\leq n_{j}\;h\left(\delta_{\tau }\right)+o\left(n_{j}\right)$, and symmetrically for *x*. Taking lim inf along $n_{j}$ bounds ${\lim\inf}_{n\to \infty }K\left(\cdot ↾n\right)/n$ from above.    □

**Remark 1.** 
*Persistent correlation for all sufficiently large n controls the upper effective dimension ${\mathrm{Dim}}_{\mathrm{eff}}$ (Proposition 3); subsequential correlation controls only the lower effective dimension $\dim_{\mathrm{eff}}$ (Proposition 4). The distinction matters because $\dim_{\mathrm{eff}}\left(x\right)\leq {\mathrm{Dim}}_{\mathrm{eff}}\left(x\right)$ in general, with equality only when the limit $\lim_{n}K\left(x↾n\right)/n$ exists.*

##### From Effective to Classical Hausdorff Dimension

Propositions 3 and 4 give a pointwise algorithmic version of the Hausdorff-dimension obstruction. The effective Hausdorff dimension is, by the Lutz–Staiger results [33,36] and the point-to-set principle [35] stated above, the individual-sequence analogue of classical Hausdorff dimension. Consequently, two algorithmically independent infinite binary sequences of sufficiently large effective dimension cannot maintain high Hamming correlation asymptotically. This justifies the Hausdorff-dimension framing of the binary-sequence theory. It does *not* prove that the LZ-compressed length of a serialised real-valued time series is a consistent estimator of Kolmogorov complexity, of effective Hausdorff dimension, or of the classical Hausdorff dimension of any underlying attractor; that empirical translation requires the calibration of Section 3, Section 4 and Section 5.

For fBm specifically, the classical graph dimension satisfies $\dim_{H}\left(\mathrm{graph}\;\mathrm{of}\;{\mathrm{fBm}}_{H}\right)=2-H$, so smaller *H* corresponds to rougher paths and larger graph dimension. The empirical compression ratio ${\tilde{C}}_{\mathrm{LZ}}$ of a finite sampled path (the normalised LZ estimator ${\tilde{C}}_{\mathrm{LZ}}$ is formally defined in Section 3.2, where the symbol-sequence generation, compression step, and normalisation are specified), however, depends on the chosen finite-resolution encoding (Section 3.2.1, Table 2). The mfBm experiments of Section 5 should therefore be read as empirical evidence consistent with the effective-dimension perspective of Propositions 3 and 4, rather than as a direct theorem about real-valued sample paths.

A time series with normalised LZ complexity ${\tilde{C}}_{\mathrm{LZ}}\left(x\right)\approx \alpha$ has its values lying on an attractor whose Hausdorff dimension is *related to* α, but the relationship is not one-to-one in general. In the binary-string setting, the Staiger [36] and Lutz [33] results directly identify the complexity rate with the effective Hausdorff dimension of the sequence. For dynamical systems, however, the connection between the entropy rate (which the normalised LZ complexity approximates) and the dimension of the attractor is mediated by the Lyapunov spectrum through the Ruelle–Pesin formula [37,38] and the Kaplan–Yorke conjecture [39]:

$$h_{\mathrm{KS}}=\sum_{\lambda_{i}>0}\lambda_{i},\;D_{\mathrm{KY}}=k+\frac{\sum_{i=1}^{k}\lambda_{i}}{|\lambda_{k+1}|},$$

where $\lambda_{1}\geq \lambda_{2}\geq \cdots$ are the Lyapunov exponents and *k* is the largest integer for which $\sum_{i=1}^{k}\lambda_{i}\geq 0$. In the special case of one-dimensional maps (such as the logistic map used in Section 4), there is a single Lyapunov exponent $\lambda_{1}=\lambda_{\max}$. In chaotic regimes with an absolutely continuous invariant measure, the entropy rate is then governed by the positive Lyapunov exponent ($h_{\mathrm{KS}}=\lambda_{\max}$, with $D_{\mathrm{KY}}=1$ as the attractor fills the interval), whereas in periodic windows the entropy is zero and the attractor is a finite set. For higher-dimensional systems, the normalised LZ complexity remains a proxy for the entropy rate $h_{\mathrm{KS}}$, but recovering the attractor dimension additionally requires knowledge of the Lyapunov spectrum. In our coupled logistic map experiment (Section 4), each uncoupled map is one-dimensional, and the coupled system is two-dimensional with a transversal Lyapunov exponent $\lambda_{T}\left(\varepsilon \right)=\ln\left|1-2\varepsilon \right|+\lambda_{\max}$ that we compute explicitly.

Two independent series lying on high-dimensional attractors must “wander” over a large region, making incidental alignment geometrically improbable.

### 2.6. Noise Increases Kolmogorov Complexity

Measuring the complexity of a time series is not entirely straightforward. One issue is that observed time series from natural sources typically contain measurement noise. In general, random noise will increase the complexity of a data object. The Posobin–Shen theorem [40] shows how it increases Kolmogorov complexity in a quantifiable way.

**Proposition 5** 
(Posobin–Shen [40])**.** *Let x be a binary string of length n whose Kolmogorov complexity satisfies C(x)≥αn, where α=H(p) for some p≤1/2 and H(·) is the binary Shannon entropy. Suppose each bit of x is independently flipped with probability τ. Then, with probability at least 1−1/n the complexity of the perturbed string satisfies (the Posobin–Shen result is stated for plain (non-prefix) Kolmogorov complexity C(x), whereas Section 2 uses prefix complexity K(x); the two quantities differ by at most O(logn) for strings of length n [16], so the asymptotic bounds carry over to the prefix setting with only sub-linear corrections)*

$$C\left(N_{\tau }\left(x\right)\right)\geq H\left(N\left(p,\tau \right)\right)\;n-o\left(n\right),$$

*where N(p,τ)=p+τ−2pτ. Moreover, for infinite sequences, the effective Hausdorff dimension increases almost surely.*

This result implies that the increase in complexity depends both on the initial complexity class of the string and on the noise level τ. In the ideal binary setting, the lower bound holds with high probability, but for finite real-valued time series, the exact complexity increase depends on the representation and noise characteristics. Consequently, empirical thresholds (such as the $J_{\mathrm{LZ}}$ threshold used later in Section 7) should be interpreted as application-dependent heuristics rather than universal constants.

If a “simple” binary string with true normalised Kolmogorov complexity $\alpha_{0}=K\left(x\right)/n\approx 0$ is corrupted by independent bit-flip noise with rate τ (say τ≈0.01–0.05 as a stylised model of measurement error), the Posobin–Shen lower bound shows that its complexity rate becomes at least H(τ) rather than zero, where *H* here is the binary Shannon entropy of τ. Numerically, H(0.01)≈0.081, H(0.02)≈0.141, H(0.05)≈0.286, so for plausible measurement-noise rates, the analytical *binary noise floor* sweeps the interval [0.08,0.29]. The upper end of this range is close to the bounded-logistic operating threshold θ=0.3, so practitioners working at appreciable noise levels should re-calibrate the threshold against surrogates of their own noise process. The empirical floor on ${\tilde{C}}_{\mathrm{LZ}}$ (the byte-level LZ ratio used throughout this paper) is a different quantity that depends on the serialisation: under pickle+float32, a noiseless periodic logistic orbit gives ${\tilde{C}}_{\mathrm{LZ}}\approx 0.011$ (Table 2), and added Gaussian noise on the same orbit raises this only modestly. The two floors should not be conflated; the empirical screening threshold θ=0.3 is a bounded-logistic operating value rather than a consequence of the binary noise bound (Section 7).

**Figure 2:**
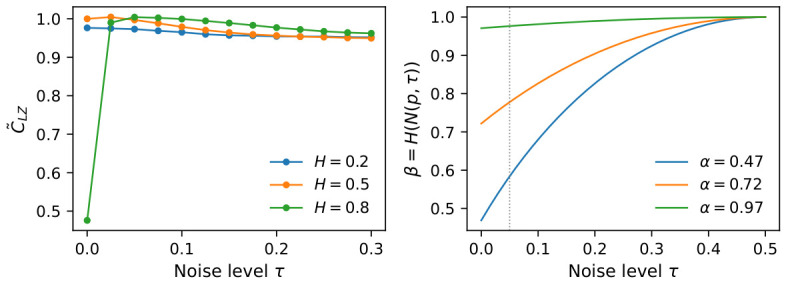
(**Left**) LZ complexity increases monotonically with noise level τ for fBm series with different Hurst parameters. (**Right**) The Posobin–Shen analytical lower bound β=H(N(p,τ)) as a function of τ for different initial complexities α=H(p). Even small noise (τ=0.05) pushes a simple series (α=0.47) to β≈0.57.

Figure 2 illustrates this numerically for fBm series and analytically for the binary case.

### 2.7. Connection to Algorithmic Causality

In this work, we are interested in how complexity can help assess the evidential weight of an observed correlation—specifically, whether it provides grounds for further causal investigation. According to the Algorithmic Markov Condition (AMC) [41], the true causal network is the one minimising Kolmogorov complexity (see also [42]). When two time series have high algorithmic mutual information I(x:y)=K(x)+K(y)−K(x,y)≫0 *and* both have individually high complexity K(x),K(y), this constitutes evidence that the correlation reflects a non-trivial relationship rather than coincidence—because independently drawn high-complexity sequences are algorithmically independent with overwhelming probability. Our complexity-filtered correlation test can thus be seen as a practical, computable *screening* step: it identifies correlations worth investigating with dedicated causal discovery methods, analogous to MDL-based approaches [10,11]. We emphasise that our framework does not, by itself, establish causation; it provides a principled criterion for distinguishing correlations that are likely meaningful from those that are likely accidental.

## 3. Complexity Estimators and the Joint Indicator $J_{\mathrm{LZ}}$

This section describes the empirical complexity estimators used throughout this paper and introduces the joint complexity indicator $J_{\mathrm{LZ}}$, the main methodological contribution. We first define the LZ-based and spectral-entropy complexity proxies together with their normalisations then address the two principal practical concerns: sensitivity to serialisation choice and sample-size requirements. We close with the screening criterion $J_{\mathrm{LZ}}$ and contrast it with the alternative similarity indicator $S_{\mathrm{LZ}}$.

### 3.1. From Binary Strings to Real-Valued Time Series

Before introducing the estimators, it is useful to make explicit the gap between the rigorous binary-string theory of Section 2 and the empirical pipeline applied to real-valued time series. We distinguish four levels:

$$\begin{array}{cc} \mathrm{Level}\;1 & \mathrm{binary}\;\mathrm{strings}\;u,v\in {\left\{0,1\right\}}^{n}\;\mathrm{and}\;\mathrm{Hamming}\;\mathrm{correlation}\;\hat{\rho };\\ \mathrm{Level}\;2 & \mathrm{finite-dimensional}\;\mathrm{real}\;\mathrm{vectors}\;x,y\in R^{N}\;\mathrm{and}\;\mathrm{Pearson}\;\mathrm{correlation}\;\rho ;\\ \mathrm{Level}\;3 & \mathrm{serialised}\;\mathrm{real-valued}\;\mathrm{time}\;\mathrm{series}\;(\mathrm{byte}\;\mathrm{strings}\;\mathrm{produced}\;\mathrm{by}\;\mathrm{float}32/\mathrm{float}64/\mathrm{int}16/\mathrm{int}8\;\mathrm{encoding});\\ \mathrm{Level}\;4 & \mathrm{compressed}\;\mathrm{byte}\;\mathrm{strings}\;\mathrm{zlib.compress}(s)\;\mathrm{used}\;\mathrm{as}\;\mathrm{empirical}\;\mathrm{proxies}\;\mathrm{for}\;K. \end{array}$$

Only Level 1 is fully rigorous in the present theory: Propositions 1 and 2 apply to binary strings under Hamming correlation, and the effective-dimension bridge of Section 2.5.1 applies to infinite binary sequences. Appendix A shows that, *for binary strings*, replacing Hamming correlation by the empirical Pearson correlation changes the counting bound only by a polynomial ${\left(n+1\right)}^{2}$ factor and therefore preserves the same exponential entropy rate $h(\delta_{\tau })$. This result does *not* establish an analogous volume bound for arbitrary real-valued vectors in $R^{N}$; the chain of rigorous coverage is

binaryHamming⟶binaryPearson⟶serialisedreal-valueddata,

and only the first two arrows are covered by the finite-string theory. Levels 3 and 4 are methodological translations: they invoke a chosen serialisation (Section 3.2.1) and a chosen lossless compressor as a computationally tractable upper bound on *K* [16], and the resulting numerical quantities depend explicitly on those choices. The transition from binary strings to real-valued time series is therefore handled empirically, through serialisation, compression, and calibration.

Throughout the remainder of this paper, the empirical $J_{\mathrm{LZ}}$ procedure should therefore be read as a *theoretically motivated, empirically calibrated diagnostic* operating at Level 4, with the rigorous guarantees confined to the binary Levels 1–2. The toy-model experiments of Section 4 and Section 5 provide the empirical calibration; the binary theorems and the effective-dimension bridge provide the conceptual motivation. We do not claim that the Level-4 compression ratio is a consistent estimator of Kolmogorov complexity, of effective Hausdorff dimension, or of the classical Hausdorff dimension of any underlying attractor.

### 3.2. Complexity Estimators

We use two primary empirical complexity measures, each a proxy for Kolmogorov complexity: LZ-based complexity and spectral entropy. We also discuss ordinal/permutation entropy as a useful robustness check, but it is not used in the reported experiments.

LZ-Based Complexity

The Lempel–Ziv (LZ) complexity provides a practical, compression-based proxy for Kolmogorov (algorithmic) complexity. Given a real-valued time series $x=\left(x_{1},\ldots ,x_{N}\right)\in R^{N}$ (we use boldface throughout to distinguish real-valued series from the binary strings $u,v\in {\left\{0,1\right\}}^{n}$ of Section 2), we proceed as follows:

1. **Symbol sequence generation.** Map x to a byte string by serialising the sequence as a float32 array via Python’s pickle protocol (pickle.dumps(x.astype(np.float32))). This yields a byte string s of approximate length M≈4N+c, where c∼32 bytes is the pickle header overhead. Alternative serialisations (raw IEEE-754 float64 via struct.pack, uniform int16 or int8 quantisation) are compared in Section 3.2.1; all main-text experiments in this paper use pickle+float32 to match the original implementation, with robustness to the alternatives reported separately.
2. **Compression-based complexity.** Compress the byte string using the LZ77-based zlib algorithm:
  $$C_{\mathrm{LZ}}\left(x\right)=\left|\mathrm{zlib}.\mathrm{compress}(s)\right|,$$
  where |·| denotes the resulting compressed length in bytes. More regular (low-complexity) series admit more repeated patterns and thus compress to shorter lengths, whereas high-complexity (e.g., chaotic) series yield larger $C_{\mathrm{LZ}}$.
3. **Normalisation.** To compare series of equal length *N*, we normalise by the original byte-string length *M*:
  $${\tilde{C}}_{\mathrm{LZ}}\left(x\right)=\frac{C_{\mathrm{LZ}}\left(x\right)}{M}\;\in \left(0,1+\epsilon \right].$$
  This ensures scale-invariant comparisons. The constant ϵ is the small fixed overhead of the compressor’s pickle and zlib headers and dictionary state; for the experiments in this paper, ϵ≲0.01, and Table 2 indeed reports values up to ${\tilde{C}}_{\mathrm{LZ}}\approx 1.00$ on incompressible inputs.

A perfectly periodic series compresses nearly to zero; a chaotic or rough series compresses poorly, giving ${\tilde{C}}_{\mathrm{LZ}}\approx 1$.

#### 3.2.1. Serialisation: Choice and Robustness

Because LZ compression operates on byte streams, the conversion from a real-valued time series to bytes is part of the method and needs to be made explicit. We consider four schemes:

- **pickle+float32.** Python’s pickle.dumps(x.astype(np.float32))— 4 bytes per value plus a small header. This is the original implementation used throughout this paper.
- **struct+float64.** Raw IEEE-754 double-precision bytes via struct.pack—8 bytes per value, no framing.
- **int16.** Uniform quantisation onto $2^{16}$ levels over the observed range, packed as 2 bytes per value.
- **int8.** Uniform quantisation onto $2^{8}=256$ levels, 1 byte per value.

Table 2 reports ${\tilde{C}}_{\mathrm{LZ}}$ under all four schemes on six canonical signal classes: bounded recurrent orbits (periodic and chaotic logistic), unbounded Gaussian increments (fGn, three Hurst values), their cumulative sums (fBm paths, three Hurst values), and uniform white noise.

**Table 2:** ${\tilde{C}}_{\mathrm{LZ}}$ under four serialisation schemes, averaged over 20–30 realisations. Logistic series use N=5000; fGn/fBm series use N=2000. Standard deviations are below 0.02 in every cell.

Signal	pickle+float32	struct+float64	int16	int8
logistic r=3.4 (periodic)	0.011	0.004	0.004	0.006
logistic r=3.9 (chaotic)	0.890	0.943	0.970	0.462
fBm path H=0.1 (rough)	0.919	0.959	0.998	0.954
fBm path H=0.5	0.913	0.955	1.003	0.972
fBm path H=0.9 (smooth)	0.897	0.937	0.990	0.497
fGn H=0.1	0.930	0.959	0.996	0.948
fGn H=0.5	0.930	0.959	0.997	0.949
fGn H=0.9	0.924	0.957	0.996	0.944
uniform noise	0.900	0.944	1.001	1.002

##### Reading the Table

Several patterns emerge:

*(1) Every serialisation separates the periodic logistic from the chaotic one.* All four schemes give ${\tilde{C}}_{\mathrm{LZ}}\approx 0.01$ at r=3.4 and a chaotic value substantially higher (ranging from 0.46 for int8 to 0.97 for int16). The bounded recurrent logistic orbit is the regime where LZ discrimination is sharpest, and results are robust across serialisation.

*(2) int8 collapses chaotic logistic but reveals fBm roughness.* int8 is the only scheme that produces a strong *H*-dependence on fBm paths: ${\tilde{C}}_{\mathrm{LZ}}$ drops from 0.954 at H=0.1 to 0.497 at H=0.9, a factor-of-two change. This is because coarse (256-level) quantisation of smooth paths produces long runs of identical bytes that zlib exploits; rough paths do not admit such runs. int8 is also, however, the only scheme that distorts the chaotic logistic case (reducing its ${\tilde{C}}_{\mathrm{LZ}}$ to 0.46). No single scheme dominates on both toy models.

*(3) Finer serialisations saturate on Gaussian-valued data.* int16, struct+float64, and pickle+float32 all give ${\tilde{C}}_{\mathrm{LZ}}\approx 0.92$–0.99 for fGn and fBm paths essentially regardless of *H*, because each sample has a nearly unique byte representation. The small trend visible in the pickle+float32 column (0.919–0.897 across H∈[0.1,0.9] for fBm paths) is the signal analysed in Section 5; it is real but modest, a ≈2% relative variation rather than the strong monotone law suggested by the theoretical Hausdorff relation $\dim_{H}=2-H$.

*(4) The path-versus-increment compression comparison is encoding-dependent.* The relative compressibility of an fBm path and its fGn increments has no universal ordering; it depends on the serialisation. Under coarse encodings such as int8, smooth (high-*H*) fBm paths can become much more compressible than their fGn increments, because the path contains long low-variation runs that the compressor exploits, whereas rough (low-*H*) paths remain close to their increments. Under fine encodings such as pickle+float32, these differences are much smaller because sample-level numerical variation dominates the byte stream (the pickle+float32 columns of Table 2 differ by only a few percent between the fBm-path and fGn rows at matching *H*). The path-versus-increment comparison should therefore not be summarised by a universal ordering. The operative point for this paper is the separate one that smooth *trends* drive the Yule nonsense-correlation phenomenon, which is handled by the stationarity pre-step rather than by the pointwise compressibility comparison.

##### Choice for the Main Experiments

For continuity with this paper’s original figures, we report main-text results using pickle+float32, the scheme under which those figures were generated. We do not claim this is optimal: for bounded recurrent dynamics, struct+float64 and int16 give cleaner contrast, and for smoothness-sensitive discrimination on Gaussian-valued paths, int8 is substantially more informative. The screening conclusion of this paper—that periodic/simple regimes produce spurious correlations frequently and chaotic/rough regimes do not—is preserved under every scheme in Table 2, because this conclusion is driven by the large periodic-versus-chaotic contrast (a gap of ∼0.9 in every column). Finer comparisons within the Gaussian-valued family are not preserved across schemes: the fine-quantisation columns (pickle+float32, struct+float64, int16) and the coarse-quantisation column (int8) can rank individual fBm/fGn rows differently, which is precisely the phenomenon motivating the open question below.

##### An Open Question: Task-Optimal Serialisation

Table 2 raises a question we pose but do not solve. The same fBm path at H=0.9 yields ${\tilde{C}}_{\mathrm{LZ}}\approx 0.99$ under int16 but ${\tilde{C}}_{\mathrm{LZ}}\approx 0.50$ under int8—a spread of roughly half the unit interval for identical underlying data. The two columns answer different questions: int16 records the individual Gaussian samples faithfully and reports that each sample is, in isolation, near-incompressible; int8 records a much coarser approximation in which the smoothness of consecutive values produces compressible runs. Which is correct depends on what one is trying to discriminate. For bounded recurrent orbits where the absolute values carry the signal (e.g., distinguishing a period-2 logistic orbit from a chaotic one), finer quantisation preserves the relevant structure. For Gaussian-valued paths where only the local difference structure carries the smoothness signal, coarse quantisation is essential—the fine quantisation destroys the signal by preserving per-sample noise that dominates the compression budget.

A principled formulation of this trade-off is the natural next step. Given a signal class S (e.g., fGn parametrised by *H*) and a discrimination task *T* (e.g., screening independent pairs for spurious correlation), one would ask for the quantisation $Q^{*}$ that maximises the mutual information between Q(x) and a sufficient statistic for *T* at a fixed description-length budget. We conjecture this is a rate–distortion problem with a task-specific distortion measure and that the optimal $Q^{*}$ interpolates between the two extremes visible in Table 2 as the signal class moves from bounded-recurrent to Gaussian-valued. Working out this correspondence—and replacing the empirical table with a closed-form prescription—is left for future work. We emphasise that the main-text experiments of this paper are internally consistent: all figures use the documented pickle+float32 pipeline, and no conclusion relies on a claim about which serialisation is globally optimal.

##### Limitations of the Pointwise Estimator for Gaussian Data

On Gaussian-valued processes (fGn, fBm at fine serialisations), the pointwise ${\tilde{C}}_{\mathrm{LZ}}$ is a weak discriminator across smoothness regimes: the compressibility is dominated by the $∼\;\log_{2}\left(N\right)$ bits per sample required to distinguish *N* Gaussian samples on a fine alphabet, and variations due to autocorrelation contribute only a small correction. Two mitigations carry information where the pointwise estimator does not:

- *Use coarser serialisation when smoothness discrimination matters.* int8 (or equivalently, symbolic dynamics via partition) reveals the *H*-dependence that finer schemes miss (Table 2).
- *Use the pairwise false-positive rate.* As demonstrated in Section 5, the rate of spurious Pearson correlations between independent pairs is a sharp function of *H* even when the pointwise ${\tilde{C}}_{\mathrm{LZ}}$ is nearly flat. This is the empirical signal that actually validates the screening framework on Gaussian data.

##### Practical Implication

For any specific data class, the practitioner should empirically calibrate by comparing ${\tilde{C}}_{\mathrm{LZ}}$ under candidate serialisations to a known null distribution at matching sample size and distribution (Step 2–3 of the recipe in Section 7). Symbolic encodings adapted to the domain (for example, the left/right partition for interval maps) often outperform uniform quantisation when domain knowledge is available [43].

##### Sample-Size Requirements

For short time series (N<100), ${\tilde{C}}_{\mathrm{LZ}}$ becomes unreliable because the compressor’s dictionary overhead dominates the compressed output. We recommend N≥500 for stable estimates; at N=500, fGn sweeps already resolve the false-positive transition in the smooth-*H* regime (Section 5).

##### Summary of Practical Choices

Table 3 collects the key recommendations.

**Table 3:** Practical recommendations for LZ complexity estimation.

Factor	Recommendation
Serialisation method	pickle+float32 (default, matches main figures); int8 for smoothness-sensitive tasks; see Section 3.2.1
Sample size *N*	N≥500 for stable ${\tilde{C}}_{\mathrm{LZ}}$; unreliable for N<100
Normalisation	By byte-string length *M* (or equivalently by *N*)
Calibration	Empirically via surrogates matched to data class and length
Indicator	$J_{\min}$ as primary; $J_{\mathrm{geom}}$ as smoother alternative (Table 4)

**Table 4:** The four power-mean candidates evaluated on representative asymmetric pairs. A pair is “flagged ✓” at a given threshold if the indicator exceeds θ=0.3. Values for the logistic pairs use N=5000 pickle+float32; fBm values use N=2000.

Pair (x,y)	${\tilde{C}}_{\mathrm{LZ}}\left(x\right)$	${\tilde{C}}_{\mathrm{LZ}}\left(y\right)$	$J_{\max}$	$J_{\mathrm{geom}}$	$J_{\mathrm{harm}}$	$J_{\min}$
logistic r=3.4 vs. r=3.9	0.011	0.890	0.890 ✓	0.099	0.022	0.011
logistic r=3.4 vs. r=3.4	0.011	0.011	0.011	0.011	0.011	0.011
logistic r=3.9 vs. r=3.9	0.890	0.890	0.890 ✓	0.890 ✓	0.890 ✓	0.890 ✓
fBm H=0.1 vs. H=0.9 (int8)	0.954	0.497	0.954 ✓	0.689 ✓	0.654 ✓	0.497 ✓

##### Fourier-Bounded Complexity (Spectral Entropy)

Justified by the Staiger [36] identification of Hausdorff dimension with the entropy of a language, we use the spectral entropy as a Fourier-domain complexity proxy. Given a real-valued time series $x=(x_{1},\ldots ,x_{N})$, we proceed through the following steps:

1. **Detrending.** Subtract the mean: ${\tilde{x}}_{n}=x_{n}-\bar{x}$, n=1,…,N. (Optionally, a linear trend can be removed and a window function applied to mitigate spectral leakage, though in our implementation we use only mean subtraction.)
2. **Discrete Fourier transform (DFT).** Compute the one-sided DFT for k=0,…,⌊N/2⌋:
  $$X_{k}=\sum_{n=1}^{N}{\tilde{x}}_{n}\;e^{-2\pi i(n-1)k/N}.$$
  (An alternative method inspired by [44] to more directly approximate complexity via DFT is discussed in Appendix C.)
3. **Power spectral density (PSD).** Form the power at each frequency: $P_{k}={\left|X_{k}\right|}^{2}$, k=0,…,⌊N/2⌋.
4. **Normalisation to a probability distribution.** Normalise $P_{k}$ so that $\sum_{k}p_{k}=1$:
  $$p_{k}=\frac{P_{k}}{\sum_{j=0}^{\left\lfloor N/2\right\rfloor }P_{j}}.$$
5. **Normalised Shannon spectral entropy.** Compute:
  $$K_{F}\left(x\right)=\tilde{H}\left(x\right)=\frac{-\sum_{k=1}^{\left\lfloor N/2\right\rfloor }p_{k}\ln p_{k}}{\ln(\lfloor N/2\rfloor )}\;\in \left[0,1\right].$$
  Note that the sum starts at k=1 and the normalising denominator uses ⌊N/2⌋ rather than ⌊N/2⌋+1: detrending in Step 1 forces $X_{0}=0$ and hence $p_{0}=0$, so only ⌊N/2⌋ bins carry mass, and the maximum attainable entropy is ln⌊N/2⌋.

A signal concentrated in one frequency (periodic) has $K_{F}\approx 0$; white noise (flat spectrum) has $K_{F}\approx 1$. This serves as a Fourier-bounded proxy for Kolmogorov complexity. The informal chain K/n≈dim≈H(powerspectrum) holds rigorously only in restricted settings (for binary infinite sequences via Staiger [36] and Lutz [33] and for one-dimensional dynamical systems where a single Lyapunov exponent suffices); for higher-dimensional dynamics, the entropy rate–dimension relationship is mediated by the Lyapunov spectrum (Section 2.5), and on Gaussian-valued data, the approximation is qualitative rather than quantitative (Section 3.2.1, Table 2).

##### Remarks on Spectral Entropy

Unlike time-domain correlation, spectral entropy captures how energy is distributed across frequencies, making it responsive to subtle synchronisation in chaotic signals even when pointwise correlation is low. The choice of natural logarithm (used consistently in both numerator and normalising denominator) ensures $\tilde{H}\in \left[0,1\right]$. Spectral entropy is closely related to the spectral flatness measure in signal processing and to the inverse participation ratio in physics.

##### Ordinal and Permutation-Based Complexity Measures

An alternative class of complexity measures uses ordinal encodings. Permutation entropy measures the Shannon entropy of ordinal patterns extracted from the time series and has been widely used as a robust complexity indicator for non-linear dynamical systems. Because ordinal methods depend only on rank ordering rather than numerical precision, they are less sensitive to floating-point representation and may serve as useful robustness checks alongside compression-based estimators such as ${\tilde{C}}_{\mathrm{LZ}}$ [43].

##### Pearson Correlation

For completeness (and since this indicator is used throughout the experiments alongside our complexity-based indicators), the empirical Pearson correlation of two real-valued series $x=(x_{1},\ldots ,x_{N})$ and $y=(y_{1},\ldots ,y_{N})$ is

(3) $$\rho \left(x,y\right)=\frac{\sum_{k=1}^{N}\left(x_{k}-\bar{x}\right)\left(y_{k}-\bar{y}\right)}{\sqrt{\sum_{k=1}^{N}{\left(x_{k}-\bar{x}\right)}^{2}}\sqrt{\sum_{k=1}^{N}{\left(y_{k}-\bar{y}\right)}^{2}}}.$$

The binary-string counterpart used in the theoretical section is given by Definition A1 in Appendix A; the two formulas coincide when real-valued entries are restricted to {0,1}.

##### Computational Cost

All complexity estimators are lightweight. For a time series of length N=5000 (our standard experimental setting), on a single core of a standard workstation (Intel i7, 3.2 GHz), LZ compression via zlib takes approximately 0.3 ms per series (including byte serialisation); spectral entropy computation takes approximately 0.8 ms (dominated by the FFT). The zlib compression step is approximately linear in the input length in the regime considered here (zlib’s DEFLATE uses a fixed-size sliding window of 32 KB and hash chains, giving amortised linear cost in *N*), while the FFT used for spectral entropy has O(NlogN) complexity.

A structural advantage worth highlighting is that $J_{\mathrm{LZ}}$ depends only on the individual marginal complexities ${\tilde{C}}_{\mathrm{LZ}}\left(x\right)$ and ${\tilde{C}}_{\mathrm{LZ}}\left(y\right)$, not on any joint quantity, so for a population of *K* candidate series, the LZ values can be *cached* per series and reused across all K(K−1)/2 pairs: only *K* compressions are needed in total, not $O(K^{2})$. The Pearson correlations themselves remain per-pair at O(N) each. Concretely, screening all pairwise correlations among K=1000 series (≈500,000 pairs) requires ≈0.3 s for all K=1000 LZ computations (cached), ≈0.8 s for all spectral entropies (cached), and the remaining cost is the ≈500,000 Pearson evaluations themselves; the complexity-related overhead is negligible relative to the correlation computation. The contrast with the Normalised Compression Distance (Section 6) is structural: NCD requires $O(K^{2})$ pairwise compressions of concatenated series and cannot be cached the same way.

### 3.3. Complexity-Similarity Indicators

#### 3.3.1. $S_{\mathrm{LZ}}$ (Complexity Similarity Only)

It might be suggested that we can measure how similar the complexities of the two series are according to:

$$S_{\mathrm{LZ}}\left(x,y\right)=-\left|{\tilde{C}}_{\mathrm{LZ}}\left(x\right)-{\tilde{C}}_{\mathrm{LZ}}\left(y\right)\right|.$$

However, while it is true that $S_{\mathrm{LZ}}=0$ when two series are equally complex, this does not capture whether they are *jointly* complex. Two simple series with identical low LZ complexity will have $S_{\mathrm{LZ}}=0$, thereby incorrectly suggesting that the high-complexity criterion is met.

#### 3.3.2. Shortlist of Candidate Joint-Complexity Indicators

To require that *both* series individually have high complexity, five natural candidates suggest themselves:

$$\begin{array}{cc} J_{\max}\;\left(x,y\right) & =\max\{{\tilde{C}}_{\mathrm{LZ}}\left(x\right),{\tilde{C}}_{\mathrm{LZ}}\left(y\right)\},\\ J_{\mathrm{geom}}\;\left(x,y\right) & =\sqrt{{\tilde{C}}_{\mathrm{LZ}}\left(x\right)\;{\tilde{C}}_{\mathrm{LZ}}\left(y\right)},\\ J_{\mathrm{harm}}\;\left(x,y\right) & =\frac{2{\tilde{C}}_{\mathrm{LZ}}\left(x\right){\tilde{C}}_{\mathrm{LZ}}\left(y\right)}{{\tilde{C}}_{\mathrm{LZ}}\left(x\right)+{\tilde{C}}_{\mathrm{LZ}}\left(y\right)},\\ J_{\min}\;\left(x,y\right) & =\min\{{\tilde{C}}_{\mathrm{LZ}}\left(x\right),{\tilde{C}}_{\mathrm{LZ}}\left(y\right)\},\\ J_{\mathrm{NCD}}\;\left(x,y\right) & =1-\mathrm{NCD}(x,y). \end{array}$$

The first four are all generalised means of the two individual complexities, satisfying min≤harm≤geom≤max by the power-mean inequality; all four collapse to ${\tilde{C}}_{\mathrm{LZ}}$ on equal-complexity pairs. The fifth ($J_{\mathrm{NCD}}$) is a fundamentally different construction based on the Normalised Compression Distance (Section 6) that detects *joint dependence* rather than *joint complexity*; we include it here only for completeness and return to it in Section 6.

#### 3.3.3. How the Four Means Differ on Asymmetric Pairs

Since all four means agree on symmetric pairs, the comparison is driven entirely by asymmetric ones. Table 4 reports the values on four representative asymmetric pairs drawn from the logistic map and fBm, together with the screening decision each indicator would make at threshold θ=0.3.

#### 3.3.4. Theoretical Guidance vs. Practical Robustness

Proposition 2 gives a formal hint: exponential suppression of spurious alignment requires $\max\left\{\alpha ,\beta \right\}>h\left(\delta_{\tau }\right)$, so the theorem’s minimal hypothesis is only that *at least one* series be sufficiently complex. That would suggest $J_{\max}$. In practice, however, the screening task is asymmetric: the false-positive risk comes from the case where one series is a simple monotone trend and the other is noisy, precisely the scenario where $J_{\max}$ is blind (row 1 of Table 4). A practical screening indicator should flag this case as untrustworthy, which rules $J_{\max}$ out.

Of the three remaining candidates (geometric mean, harmonic mean, min), all three correctly flag the asymmetric logistic pair as simple. They differ mainly in how aggressively they penalise moderate asymmetry: the geometric mean is the gentlest, the harmonic mean intermediate, and the min the most conservative. Which is preferable depends on the user’s trade-off between false positives and false negatives. On this paper’s toy models, these three indicators give identical screening decisions at every threshold we consider:

- On the r=3.9 chaotic coupling sweep, $|J_{\min}-J_{\mathrm{geom}}|<0.0004$ throughout.
- On the independent-pair false-positive sweep r∈[2.8,3.99], the three means agree to within 0.003 at every *r*.
- On the fBm (H=0.1,H=0.9) asymmetric pair under int8 quantisation (row 4), the three means disagree substantially in magnitude but all three screen the pair as “trustworthy” at θ=0.3.

#### 3.3.5. Our Recommendation: $J_{\min}$ as Primary, $J_{\mathrm{geom}}$ as Principled Alternative

Following Occam’s razor, we adopt

$$J_{\mathrm{LZ}}\left(x,y\right)\;=\;\min\left\{{\tilde{C}}_{\mathrm{LZ}}\left(x\right),\;{\tilde{C}}_{\mathrm{LZ}}\left(y\right)\right\}\;\in \;\left[0,1\right]$$

as the primary joint complexity indicator throughout the remainder of this paper. It is the simplest of the three non-pathological candidates, the most conservative (strictly underestimates the geometric mean), and gives identical screening decisions to the other two on all experiments in this paper. Readers who prefer a smoother indicator with the same operational behaviour may substitute $J_{\mathrm{geom}}$ without changing any conclusion, as reported alongside $J_{\min}$ for representative pairs in Table 4.

#### 3.3.6. What Not to Do

As a cautionary counterexample, an early draft considered the indicator $\min({\tilde{C}}_{\mathrm{LZ}}\left(x\right),$${\tilde{C}}_{\mathrm{LZ}}\left(y\right))-|{\tilde{C}}_{\mathrm{LZ}}\left(x\right)-{\tilde{C}}_{\mathrm{LZ}}\left(y\right)|$, which combines the $J_{\min}$ floor with an asymmetry penalty. This is pathological: a pair with complexities (0.9,0.5) scores 0.1—*lower* than two uniformly simple series at (0.3,0.3) which score 0.3—directly contradicting the design goal. Any indicator that penalises asymmetry *in addition to* taking a low aggregate of the two complexities will have this failure mode; the generalised means are the natural family because they respect the ordering constraint min≤J≤max.

**Remark 2.** 
*Note the distinction between $J_{\mathrm{LZ}}$ and $S_{\mathrm{LZ}}$. The  joint complexity indicator $J_{\mathrm{LZ}}\left(x,y\right)$ is a screening criterion evaluated before inspecting a reported correlation: a pair is flagged as complexity-trustworthy only if $J_{\mathrm{LZ}}>\theta$ for some empirically chosen threshold θ. The  similarity indicator $S_{\mathrm{LZ}}\left(x,y\right)=-\left|{\tilde{C}}_{\mathrm{LZ}}\left(x\right)-{\tilde{C}}_{\mathrm{LZ}}\left(y\right)\right|$ instead measures whether the two series converge in complexity as a function of coupling strength ε and is the quantity plotted in the numerical experiments of Section 4. The two serve entirely different purposes.*

#### 3.3.7. Interpretation Table

The joint indicator $J_{\mathrm{LZ}}$ should be read together with the magnitude of the Pearson correlation. Table 5 summarises the intended interpretation.

A high-$J_{\mathrm{LZ}}$ pair with high |ρ| is therefore not a causal conclusion: it merely survives the low-complexity spurious-correlation screen. Common drivers, confounding, non-stationarity, and model misspecification must still be addressed by domain-specific analysis or causal-discovery methods.

**Figure 3:**
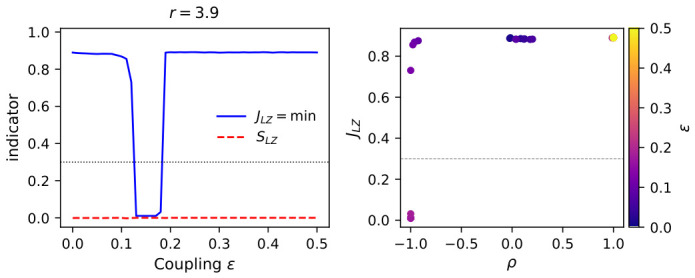
Joint-complexity screening on coupled logistic maps with r=3.9 under pickle+float32 serialisation (N=5000). (**Left**) $S_{\mathrm{LZ}}$ (red dashed) and $J_{\mathrm{LZ}}=\min\left\{{\tilde{C}}_{\mathrm{LZ}}\left(x\right),{\tilde{C}}_{\mathrm{LZ}}\left(y\right)\right\}$ (blue solid) as functions of the coupling strength ε. At ε=0, the two maps are independent and individually complex, giving $J_{\mathrm{LZ}}\approx 0.89$. Near the pre-synchronisation transition window ε∈[0.13,0.18], both marginal complexities collapse from approximately 0.89 to approximately 0.01, and hence $J_{\mathrm{LZ}}$ drops sharply. After full synchronisation, around $\varepsilon_{c}\approx 0.19$, the maps again trace a common chaotic orbit and $J_{\mathrm{LZ}}$ returns to approximately 0.89. In contrast, $S_{\mathrm{LZ}}$ remains close to zero throughout because the two marginal complexities track one another closely; it therefore detects similarity of complexity levels but not whether the shared complexity is high or low. (**Right**) scatter plot of $\left(\rho ,J_{\mathrm{LZ}}\right)$ coloured by ε. Weak coupling produces low-correlation, high-complexity pairs; strong coupling produces high-correlation, high-complexity pairs; and the transition window produces low-$J_{\mathrm{LZ}}$, intermediate-to-high-correlation points. The red dashed lines mark the reference screening thresholds $J_{\mathrm{LZ}}=\theta =0.3$ and |ρ|=0.5.

Figure 3 illustrates the distinction between the similarity score $S_{\mathrm{LZ}}$ and the joint screening score $J_{\mathrm{LZ}}$ on coupled logistic maps.

## 4. Experiment 1: Coupled Logistic Maps

This section provides empirical validation on a fully controlled toy model: two symmetrically coupled logistic maps. The logistic parameter *r* tunes each map’s complexity continuously from periodic (low *r*) to chaotic (high *r*), while the coupling ε sets the true degree of dependence—making it the ideal benchmark for testing whether $J_{\mathrm{LZ}}$ correctly separates spurious from genuine correlations. We report three interlocking results: (i) complexity along the bifurcation diagram tracks the Lyapunov exponent; (ii) false-positive rate of Pearson correlation is determined almost entirely by complexity and not by ε; and (iii) $J_{\mathrm{LZ}}$ detects a pre-synchronisation collapse of individual complexity just before the transition coupling $\varepsilon_{c}$.

### 4.1. Coupled Logistic Map Model

We use two symmetrically diffusively coupled logistic maps:

(4) $$\begin{array}{cc} x_{n+1} & =\left(1-\varepsilon \right)\;r\;x_{n}\left(1-x_{n}\right)+\varepsilon \;r\;y_{n}\left(1-y_{n}\right), \end{array}$$

(5) $$\begin{array}{cc} y_{n+1} & =\left(1-\varepsilon \right)\;r\;y_{n}\left(1-y_{n}\right)+\varepsilon \;r\;x_{n}\left(1-x_{n}\right), \end{array}$$

with $x_{0}=0.1$, $y_{0}=0.2$, transient of 1000 iterates discarded, N=5000 recorded. The coupling ε∈[0,0.5] determines the strength of correlations between the two series. We use two dynamical regimes, one of low and one of high complexity:

- r=3.4: period-2 orbit (low complexity, ${\tilde{C}}_{\mathrm{LZ}}\approx 0.011$);
- r=3.9: fully developed chaos (high complexity, ${\tilde{C}}_{\mathrm{LZ}}\approx 0.894$).

The logistic parameter r∈[0.0,4.0] determines the complexity of the time series. For values r≲3.5, the series are simple, while for most larger values of *r*, the series is chaotic and complex. Figure 3 illustrates the contrast between $S_{\mathrm{LZ}}$ and $J_{\mathrm{LZ}}$ on the r=3.9 chaotic coupled maps. $J_{\mathrm{LZ}}\approx 0.89$ at ε=0, correctly identifying the independently generated chaotic series as jointly complex; $S_{\mathrm{LZ}}\approx 0$ throughout and is therefore uninformative about the absolute level of complexity. The right panel confirms that high $J_{\mathrm{LZ}}$ correctly tags pairs as “genuine relationship” candidates once ρ also becomes large.

### 4.2. Complexity Along the Bifurcation Diagram

**Figure 4:**
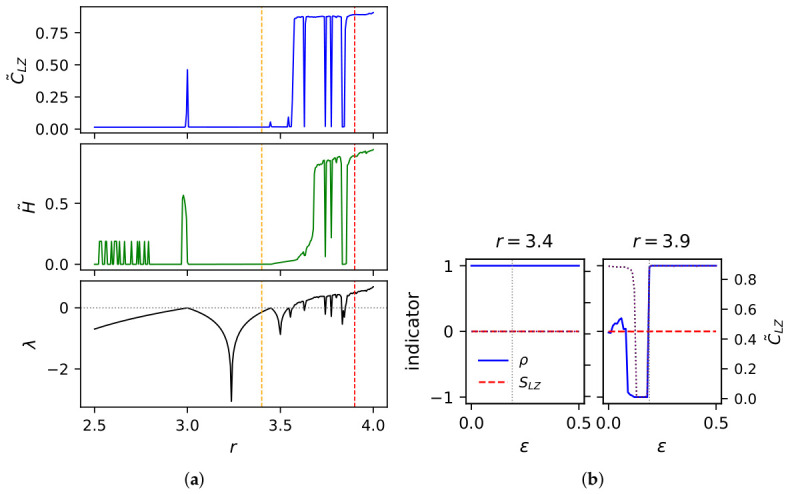
Coupled logistic maps: complexity structure and synchronisation. (**a**) Complexity and the Lyapunov exponent along the bifurcation diagram. (**b**) Synchronisation indicators versus coupling. (**a**) Normalised LZ complexity ${\tilde{C}}_{\mathrm{LZ}}$ (**top**; compressed length divided by original byte length), spectral entropy $\tilde{H}$ (**middle**), and Lyapunov exponent (**bottom**) vs. logistic parameter *r*. Complexity grows with chaos and tracks the Lyapunov exponent. For the one-dimensional logistic map in the chaotic regime, the entropy rate equals the positive Lyapunov exponent ($h_{\mathrm{KS}}=\lambda_{\max}$), so ${\tilde{C}}_{\mathrm{LZ}}$ and $\tilde{H}$ are useful proxies for entropy-rate/algorithmic-complexity behaviour. The connection to attractor dimension is indirect and, for higher-dimensional dynamical systems, additionally depends on the full Lyapunov spectrum (Section 2.5). (**b**) Synchronisation indicators vs. coupling ε for r=3.4 (**left**) and r=3.9 (**right**). The secondary axis (right side of each panel, dotted lines) shows the individual LZ complexity values ${\tilde{C}}_{\mathrm{LZ}}\left(x\right),{\tilde{C}}_{\mathrm{LZ}}\left(y\right)$. In both regimes, the two individual complexities track one another almost exactly, so $S_{\mathrm{LZ}}=-\left|{\tilde{C}}_{\mathrm{LZ}}\left(x\right)-{\tilde{C}}_{\mathrm{LZ}}\left(y\right)\right|$ stays flat at zero throughout. In the chaotic regime, both individual complexities collapse together symmetrically in a narrow window around ε≈0.14, just before the synchronisation threshold $\varepsilon_{c}\approx 0.19$ marked by the vertical dotted line; this joint collapse is invisible to $S_{\mathrm{LZ}}$ but visible in $J_{\min}=\min\left\{{\tilde{C}}_{\mathrm{LZ}}\left(x\right),{\tilde{C}}_{\mathrm{LZ}}\left(y\right)\right\}$ (Figure 3).

Figure 4a shows LZ complexity, spectral entropy, and Lyapunov exponent as functions of the logistic parameter *r*. Complexity and the Lyapunov exponent rise together: positive λ marks chaos, and both ${\tilde{C}}_{\mathrm{LZ}}$ and $\tilde{H}$ jump from near zero to near one as *r* crosses the period-doubling cascade. The vertical lines at r=3.4 and r=3.9 mark our two experimental regimes.

### 4.3. Synchronisation Indicators vs. Coupling

Figure 4b shows ρ and $S_{\mathrm{LZ}}$ as functions of ε for both regimes. The secondary *y*-axis shows the individual LZ complexity values ${\tilde{C}}_{\mathrm{LZ}}\left(x\right)$ and ${\tilde{C}}_{\mathrm{LZ}}\left(y\right)$, which are crucial for interpreting $S_{\mathrm{LZ}}$ in context.

A key empirical observation is that the two individual LZ complexities track one another almost exactly throughout the entire ε sweep, in both the periodic and the chaotic regimes: the maximum $|{\tilde{C}}_{\mathrm{LZ}}\left(x\right)-{\tilde{C}}_{\mathrm{LZ}}\left(y\right)|$ over our sweep is ≲0.001. As a consequence, $S_{\mathrm{LZ}}=-\left|{\tilde{C}}_{\mathrm{LZ}}\left(x\right)-{\tilde{C}}_{\mathrm{LZ}}\left(y\right)\right|$ is essentially flat at zero everywhere, including across the synchronisation transition. This matters because it falsifies a tempting interpretation of the pre-synchronisation phenomenon: the dip we observe is *not* an asymmetric event in which one trajectory collapses onto a periodic attractor while the other remains chaotic (which would drive $S_{\mathrm{LZ}}$ to ∼−0.89). Instead, both trajectories simplify together *symmetrically* as the system is forced toward a common simpler attractor; both ${\tilde{C}}_{\mathrm{LZ}}$ values fall together while their difference stays near zero.

The early-warning signature is therefore visible in $J_{\min}=\min\left\{{\tilde{C}}_{\mathrm{LZ}}\left(x\right),{\tilde{C}}_{\mathrm{LZ}}\left(y\right)\right\}$ but not in $S_{\mathrm{LZ}}$: as shown in Figure 3 (left), $J_{\min}$ drops sharply from ≈0.89 to ≈0.01 in a window around ε≈0.14, just before the synchronisation threshold $\varepsilon_{c}\approx 0.19$, signalling the imminent collapse of joint complexity. Since $S_{\mathrm{LZ}}$ measures only the *difference* of complexities and the collapse is symmetric, $S_{\mathrm{LZ}}$ records nothing. This is a concrete demonstration of why the level-based $J_{\min}$ is the operationally useful indicator and the difference-based $S_{\mathrm{LZ}}$ is not.

#### Synchronisation Threshold

For the chaotic regime, there exists a *critical coupling* $\varepsilon_{c}$ above which the two maps achieve full synchronisation (i.e., $x_{n}=y_{n}$ for all *n* after transients). This threshold can be predicted from the transversal Lyapunov exponent. Define

$$\lambda_{T}\left(\varepsilon \right)=\ln\left|1-2\varepsilon \right|+\lambda_{\max},$$

where $\lambda_{\max}\approx 0.496$ is the maximal Lyapunov exponent of the uncoupled logistic map at r=3.9. Full synchronisation ($x_{n}=y_{n}$ for all *n*) occurs when $\lambda_{T}\left(\varepsilon_{c}\right)=0$, giving

$$\varepsilon_{c}=\frac{1-e^{-\lambda_{\max}}}{2}\approx 0.19.$$

Below $\varepsilon_{c}$, Pearson ρ remains near zero despite genuine coupling—this is the regime where complexity-based indicators provide early warning of synchronisation.

### 4.4. False Positive Rate vs. Complexity

The core claim of this paper is that simple series produce more spurious correlations. We verify this directly: for each r∈[2.8,3.99], we generate 200 independent pairs (different initial conditions, ε=0) and record the fraction with |ρ|>0.3, i.e., correlations of non-trivial magnitude.

**Figure 5:**
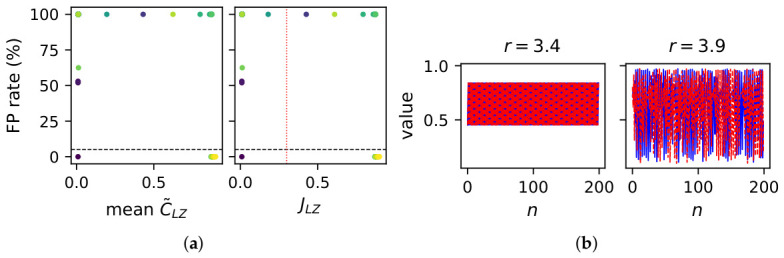
Coupled logistic maps: false positives and sample trajectories. (**a**) False-positive rate versus complexity. (**b**) Representative short time series at weak coupling. (**a**) False-positive rate (fraction of independent pairs with |ρ|>0.3) vs. mean LZ complexity (**left**) and vs. $J_{\mathrm{LZ}}=\min$ (**right**), coloured by *r*. The dashed horizontal line marks the 5% significance level; the dotted vertical line in the right panel marks the screening threshold θ=0.3. At r=3.4 (periodic, ${\tilde{C}}_{\mathrm{LZ}}\approx 0.011$–0.02): false-positive rate ≈100%. At r≥3.7 (chaotic, ${\tilde{C}}_{\mathrm{LZ}}≳0.87$): false-positive rate ≈0%. The transition is essentially bimodal: the logistic map has a sharp chaos onset near r≈3.57, so there is very little intermediate ${\tilde{C}}_{\mathrm{LZ}}$ population, and FP rate drops from 100% to 0% over a narrow window in *r*. Points in the mid-range ${\tilde{C}}_{\mathrm{LZ}}∼0.78$–0.86 correspond to periodic windows embedded in the chaotic region (e.g., the period-3 window near r=3.83) and show intermediate FP rates of 60–75%. (**b**) First 200 iterates of $x_{n}$ (blue) and $y_{n}$ (red dashed) at ε=0.02. (**Left**) r=3.4 (periodic). (**Right**) r=3.9 (chaotic). Both series at r=3.9 look independent despite genuine coupling, illustrating why LZ-based early warning is valuable.

**Figure 6:**
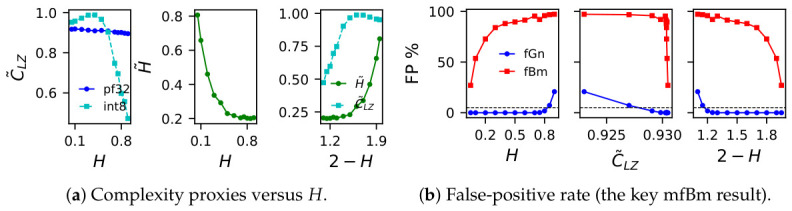
fBm complexity and the central false-positive result. (**a**) LZ complexity (**left**), spectral entropy (**centre**), and both vs. Hausdorff dimension (**right**) on fBm paths as functions of the Hurst parameter *H*. Spectral entropy decreases monotonically as *H* increases, tracking the decrease in theoretical dimension $\dim_{H}=2-H$. ${\tilde{C}}_{\mathrm{LZ}}$ under pickle+float32 shows the same monotone trend but with a much smaller magnitude; finer serialisations (struct+float64, int16) saturate near the compression limit and make the trend invisible, while coarser serialisation (int8) recovers it with a larger range (Table 2). See Section 3.2.1 for discussion. (**b**) False-positive rate (|ρ|>0.1) for independent pairs of fGn (blue) and fBm paths (red), measured over 500 independent trials at each of 12 values of H∈[0.05,0.90] (N=2000 per realisation, pickle+float32 serialisation). (**Left**) vs. Hurst *H*. (**Centre**) vs. mean ${\tilde{C}}_{\mathrm{LZ}}$. (**Right**) vs. the associated fBm path dimension 2−H (used as a calibration coordinate, not as a theorem about fGn increments). The fGn curve empirically supports the qualitative prediction: rough/less persistent stationary series essentially never produce spurious correlations at |ρ|>0.1 (FP rate ≤1.8% for H≤0.80), while smooth/persistent series do so increasingly often as H→1 (7.2% at H=0.85, 20.8% at H=0.90). The 5% threshold is crossed around H≈0.83 (equivalently, for the associated fBm path, 2−H≈1.17), as read off the *left* panel. The fBm paths (red) are non-stationary and produce spurious correlations in 27–97% of trials regardless of *H*, replicating the classical Yule nonsense-correlation phenomenon. This underscores that the complexity diagnostic must be applied only after ensuring stationarity; see Remark 3. In the centre panel, the fGn points cluster near ${\tilde{C}}_{\mathrm{LZ}}\approx 0.93$ for all *H* (the pointwise estimator saturates on Gaussian-valued data under pickle+float32, as shown in Table 2); the false-positive transition is therefore better read off the left panel (as a function of *H*) than off the centre panel (as a function of ${\tilde{C}}_{\mathrm{LZ}}$). At the smooth end of the grid, the Davies–Harte exact-circulant method of [47] fails to produce a positive-semidefinite embedding for H≥0.95 at N=2000, so we restrict the grid to H≤0.90; the trend is monotonically increasing throughout.

The result is stark (Figure 5a): periodic series *always* spuriously correlate because their symbolic itinerary is low-complexity (a finite, eventually periodic orbit), whereas chaotic series *never* spuriously correlate because they have positive entropy and generate high-complexity symbolic itineraries, even though the state space is one-dimensional. The logistic map’s own bimodal complexity distribution—${\tilde{C}}_{\mathrm{LZ}}\approx 0.02$ in the periodic regime and ${\tilde{C}}_{\mathrm{LZ}}\approx 0.88$ in the chaotic regime, with almost no values in between except in narrow periodic windows—means the 5% crossover is not resolved by this model; it occurs somewhere inside the narrow bifurcation at r≈3.57. The screening threshold θ=0.3 adopted in Section 7 is a conservative operating point for this bounded logistic setting: it assigns all periodic pairs to "screen out" and all chaotic pairs to "screen in". The smoother fGn experiment (Figure 6b) provides the complementary family-level false-positive calibration as a function of memory/smoothness.

### 4.5. Short Time Series Examples

Figure 5b shows representative time series at ε=0.02 for both regimes, with complexity and correlation statistics annotated. The periodic case (r=3.4) shows perfectly synchronised square-wave-like orbits even at very weak coupling; the chaotic case (r=3.9) shows independent-looking wandering despite the same ε.

### 4.6. Summary of Experiment 1

The coupled logistic map experiment provides clean, controlled evidence for this paper’s central claim, because the complexity of each series can be tuned continuously via *r* while the true coupling strength ε is known exactly. Three interlocking results emerge.

#### 4.6.1. Complexity Determines False-Positive Rate, Not Coupling

The most direct test is the false-positive experiment (Section 4, Figure 5a): independent pairs (ε=0) at r=3.4 produce spurious correlations |ρ|>0.3 in *100%* of trials, whereas independent pairs at r≥3.7 produce zero false positives. The transition is monotone in ${\tilde{C}}_{\mathrm{LZ}}$ but not smoothly resolved: the logistic map’s chaos onset at r≈3.57 is sharp, so ${\tilde{C}}_{\mathrm{LZ}}$ jumps from ≈0.02 to ≈0.88 across a narrow *r*-window and the 5% crossing is contained within it. The underlying reason is dynamical: periodic orbits have a low-complexity symbolic description (a finite, eventually periodic set of values), so any two period-*k* orbits with similar *r* are trivially correlated regardless of whether they share any causal connection. Chaotic orbits, by contrast, have positive entropy and produce high-complexity, effectively aperiodic symbolic itineraries that cannot accidentally align, even though the logistic state space is itself one-dimensional. The fGn experiment of Section 5 resolves the smooth-process false-positive transition more precisely as a function of *H*.

#### 4.6.2. $J_{\mathrm{LZ}}$ Captures Joint Complexity; $S_{\mathrm{LZ}}$ Does Not

A subtler finding concerns what happens when the two maps *are* genuinely coupled. $S_{\mathrm{LZ}}$ measures the *difference* of individual complexities, which is nearly zero for both regimes (the two maps always have similar ${\tilde{C}}_{\mathrm{LZ}}$) and is therefore uninformative about whether that shared complexity is high or low. The $J_{\mathrm{LZ}}=\min\left\{{\tilde{C}}_{\mathrm{LZ}}\left(x\right),{\tilde{C}}_{\mathrm{LZ}}\left(y\right)\right\}$ instead measures the *level* of joint complexity: it reads ≈0.89 for the independent chaotic maps at ε=0, correctly tagging them as jointly complex; it reads ≈0.01 for the periodic maps, correctly flagging their correlation as unreliable. The right panel of Figure 3 makes this concrete: the $\left(J_{\mathrm{LZ}},\rho \right)$ scatter cleanly separates into the correct quadrants.

#### 4.6.3. Complexity Collapses at the Synchronisation Transition

A physically interesting side-effect of the $J_{\mathrm{LZ}}$ is that it detects the synchronisation transition in the chaotic regime. Just before the critical coupling $\varepsilon_{c}\approx 0.19$, the two maps are being forced toward a common simpler attractor; in this brief window, *both* individual complexities ${\tilde{C}}_{\mathrm{LZ}}\left(x\right)$ and ${\tilde{C}}_{\mathrm{LZ}}\left(y\right)$ drop together from ≈0.89 to ≈0.01, while their difference (and hence $S_{\mathrm{LZ}}$) stays near zero. Because $J_{\mathrm{LZ}}=\min$ tracks the level of joint complexity rather than its asymmetry, it dips sharply to ≈0 across this window. Once full synchronisation is achieved ($\varepsilon >\varepsilon_{c}$), the two maps again trace a common high-complexity chaotic orbit, and $J_{\mathrm{LZ}}$ recovers to ≈0.89. This dip is a genuine pre-synchronisation warning visible in $J_{\mathrm{LZ}}$ but invisible in $S_{\mathrm{LZ}}$ (which is flat at zero throughout, since both complexities move together), providing a concrete illustration of why the joint screening criterion is more informative than the difference-based one.

#### 4.6.4. Practical Takeaway

When reporting a correlation between two time series, the first question to ask is not whether |ρ| is large but whether $J_{\mathrm{LZ}}$ is large. A high correlation among high-complexity series (top-right quadrant of Figure 3) is substantially *less likely to be explained solely by shared low-complexity structure* and is therefore worth investigating further—though it does not, by itself, establish causation or rule out common drivers, confounding, or model misspecification. The same correlation among low-complexity series (bottom-left quadrant) is essentially uninformative: simple series are, by Solomonoff’s prior, exponentially more likely to align by chance, and the logistic map experiment confirms this quantitatively.

#### 4.6.5. Uncertainty Quantification

The false-positive rates reported in Figure 5a are empirical proportions from 200 independent pairs per *r*-value. Treating each ε=0 pair as a Bernoulli trial, the Wilson 95% confidence intervals at the two endpoints of the curve are (98.2%,100.0%) at r=3.4 (observed FP rate 100%) and (0.0%,1.8%) at r=3.9 (observed FP rate 0%); the separation of the two regimes is therefore not in doubt within these intervals. In the narrow transition window r∈[3.57,3.85], the periodic windows embedded in the chaotic region (e.g., the period-3 window at r≈3.83) produce intermediate FP rates of 60–75% with 95% Wilson CIs roughly ±7 percentage points wide; the qualitative transition shape is robust under these intervals.

## 5. Experiment 2: Multivariate Fractional Brownian Motion

The multivariate fractional Brownian motion (mfBm) [45] provides a second toy model, complementary to the coupled logistic maps of Section 4. It is analytically tractable in a different way: the Hurst parameter *H* directly controls the roughness and classical Hausdorff dimension of the paths ($\dim_{H}=2-H$), giving a continuous family decoupled from any coupling parameter. We use this structure to (i) compare finite-resolution complexity proxies with the theoretical Hausdorff dimension on fBm paths (the signal is modest under fine serialisation and much sharper under coarser encodings, as reported below); (ii) demonstrate the Yule nonsense-correlation phenomenon for smooth (high-*H*) fBm paths and its sharp dependence on path roughness; and (iii) calibrate false-positive risk as a function of the memory/smoothness family.

### 5.1. Model

A *p*-multivariate fractional Brownian motion (mfBm) with Hurst parameters $H=\left(H_{1},\ldots ,H_{p}\right)\in {\left(0,1\right)}^{p}$ is the unique Gaussian, *H*-self-similar process with stationary increments:

$$\left(X_{1}\left(\lambda t\right),\ldots ,X_{p}\left(\lambda t\right)\right)\overset{\mathrm{fidi}}{=}\left(\lambda^{H_{1}}X_{1}\left(t\right),\ldots ,\lambda^{H_{p}}X_{p}\left(t\right)\right).$$

Its covariance structure is given by Proposition A1 in Appendix B. The increments of each component, the *fractional Gaussian noise* (fGn), are a stationary process with covariance $\gamma \left(k\right)=\frac{1}{2}\left({\left|k-1\right|}^{2H}-{2|k|}^{2H}+{\left|k+1\right|}^{2H}\right)$.

#### Key Property: Hausdorff Dimension

The graph of $X_{i}$ (as a subset of $R^{2}$) has Hausdorff dimension $\dim_{H}\left(\mathrm{graph}\;\mathrm{of}\;X_{i}\right)=2-H_{i}$ [46]. Thus:

- $H_{i}\approx 0$ (rough, highly irregular): $\dim_{H}\approx 2$, high LZ complexity.
- $H_{i}\approx 1$ (smooth, trending): $\dim_{H}\approx 1$, low LZ complexity, behaves like a random walk.

This dimension statement is about the fBm *path graph*. The stationary fGn increments inherit the same Hurst parameter as a memory/smoothness coordinate, but they are not themselves governed by the graph-dimension identity $\dim_{H}=2-H$. We therefore use *H* in two related but distinct ways: as a Hausdorff-dimension coordinate for fBm paths, and as a family parameter for calibrating false-positive risk in stationary fGn. The empirical prediction is that spurious correlation rates should decrease as *H* decreases, i.e., as the associated paths become rougher and the stationary increments become less smooth/less persistent.

We simulate fGn exactly using the Wood–Chan circulant embedding algorithm [47]. All mfBm experiments use N=2000 time steps and 500 independent trials per Hurst value, with the Hurst grid H∈{0.05,0.10,0.20,0.30,0.40,0.50,0.60,0.70,0.75,0.80,0.85,0.90}.

### 5.2. Complexity Proxies on fBm Paths

A first question is whether the pointwise complexity proxies track the theoretical Hausdorff dimension $\dim_{H}=2-H$ for fBm. The prediction is that rough paths (*H* small, $\dim_{H}$ close to 2) should have higher ${\tilde{C}}_{\mathrm{LZ}}$ than smooth paths (*H* close to 1, $\dim_{H}$ close to 1). We find this prediction *qualitatively* confirmed but quantitatively modest under this paper’s main serialisation (pickle+float32): ${\tilde{C}}_{\mathrm{LZ}}$ on fBm paths (N=2000) decreases from ≈0.919 at H=0.1 to ≈0.897 at H=0.9—a monotone but shallow trend of about 0.02 across the full roughness range. Spectral entropy $\tilde{H}$ shows a larger and more monotone decrease over the same range. Figure 6a presents both.

#### 5.2.1. The Signal Sharpens Under Coarser Serialisation

The modesty of the ${\tilde{C}}_{\mathrm{LZ}}$-versus-*H* signal under pickle+float32 is an artefact of the serialisation rather than of the data: on the same fBm paths, int8 quantisation reveals a much larger range (Table 2, 0.954 at H=0.1 down to 0.497 at H=0.9) because coarse quantisation of smooth paths produces long runs of identical bytes that LZ exploits. Symbolic encodings adapted to the specific dynamics—the classical left/right partition for interval maps, partition-based encodings for attractors—typically give even sharper discrimination. We keep pickle+float32 in the main figures for continuity with this paper’s original implementation and note the serialisation-dependence as a feature rather than a flaw.

#### 5.2.2. Why the Framework Still Works

The screening argument in this paper rests on two separable claims: (i) in the binary-string model, high Kolmogorov complexity suppresses spurious Hamming/Pearson alignment under the hypotheses of Proposition 2; and (ii) empirically, smooth or persistent real-valued series produce many spurious correlations while rougher stationary series produce few. Claim (ii) is the operationally testable one for fGn/fBm data, and it is a statement about *pairs* of series rather than individual compression values. As Section 5.3 shows, the false-positive rate under independent-pair fGn is a sharp function of *H* at N=2000, 500 trials per Hurst (going from ≤1.8% at H≤0.80 to 7.2% at H=0.85 and 20.8% at H=0.90) even when the pointwise ${\tilde{C}}_{\mathrm{LZ}}$ scale is compressed. The pointwise estimator is a practical proxy when the encoding has useful dynamic range; in Gaussian-increment families where it saturates, the pairwise Pearson false-positive curve is the empirical calibration target.

### 5.3. False Positive Rate: The Key mfBm Result

Figure 6b shows the main result for the mfBm toy model. For stationary fGn increments at N=2000 over 500 trials per Hurst (with Wilson 95% confidence intervals computed on the binomial proportion):

- H=0.05–0.80 (rough to mildly smooth; associated fBm path dimension 2−H>1.2): false positive rate ≤1.8% (CI upper bound ≤3.4%; essentially zero for H≤0.70).
- H=0.85: false positive rate ≈7.2% (95% CI [5.2%,9.8%]; just past the 5% threshold).
- H=0.90: false positive rate ≈20.8% (95% CI [17.5%,24.6%]).

The separation between rough and smooth regimes is therefore not in doubt within these intervals. For the non-stationary fBm paths, false-positive rates are uniformly high (27–97%) across all *H*—this is exactly the *Yule nonsense-correlation phenomenon* [6,7]: integrated non-stationary series spuriously correlate regardless of their roughness. Note that the LZ complexity diagnostic alone does *not* flag this case: under pickle+float32, fBm paths have ${\tilde{C}}_{\mathrm{LZ}}\approx 0.90$, slightly below the corresponding fGn (≈0.93) but within a window narrow enough that the pointwise complexity does not cleanly separate stationary from non-stationary regimes (Table 2). The appropriate response is the two-stage pipeline of Remark 3: first test for stationarity and difference if needed, then apply the complexity screen to the stationary residual.

**Remark 3** 
(Stationarity and complexity are distinct diagnostics)**.** *It is important to distinguish the roles of stationarity and complexity. The fBm paths illustrate that non-stationarity (specifically, integrated processes with stochastic trends) can produce spurious correlations regardless of the roughness of the increments. The pointwise LZ complexity does not cleanly separate fBm paths from their fGn increments under pickle+float32 serialisation (Table 2): both are Gaussian-valued and saturate the estimator near ${\tilde{C}}_{\mathrm{LZ}}\approx 0.90$–0.93. The practical implication is that our framework should be applied in two stages: (1) test for stationarity (e.g., ADF test [9]) and first-difference if needed; (2) apply the complexity diagnostic to the stationary residuals. The fGn results (Figure 6b) support the qualitative prediction that stationary rough/less persistent series produce fewer false-positive correlations than smooth/persistent ones. The formal guarantees of Section 2, however, remain binary-string results; the real-valued compression workflow is an empirically calibrated diagnostic in this setting.*

**Figure 7:**
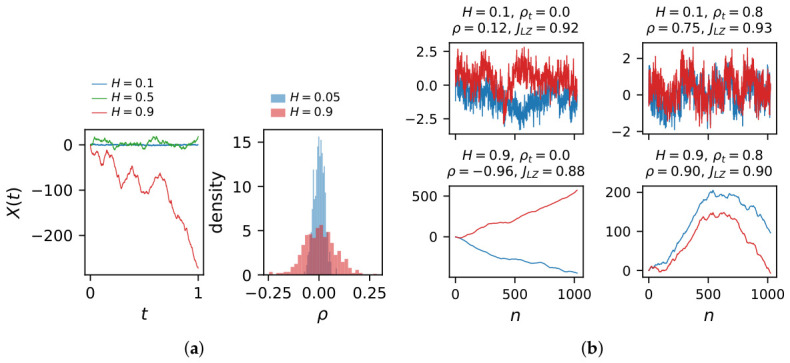
fGn roughness, correlation distributions, and bivariate regimes. (**a**) Sample paths and Pearson-ρ distributions for rough versus smooth fGn. (**b**) Four regimes of bivariate fBm. ((**a**) **Left**) fGn sample paths for different *H* (rougher = lower *H* = less persistent; for the associated fBm path, H=0.1 corresponds to a rough graph with dimension 2−H=1.9, while H=0.9 corresponds to a smoother graph with dimension 2−H=1.1). (**Right**) distribution of Pearson ρ for 500 independent fGn pairs. Smooth series (H=0.90, red) have a wide distribution with many spurious correlations; rough series (H=0.05, blue) are tightly concentrated near zero. (**b**) Four parameter regimes of bivariate fBm (N=1024 per panel, an illustrative length distinct from the N=2000 used in the quantitative false-positive sweep of Figure 6b). (**Top row**) rough (H=0.1). (**Bottom row**) smooth (H=0.9). (**Left column**) uncorrelated target ($\rho_{\mathrm{target}}=0$). (**Right column**) correlated target ($\rho_{\mathrm{target}}=0.8$). Each panel annotates the realised $\rho_{\mathrm{obs}}$ and $J_{\mathrm{LZ}}=\min\left\{{\tilde{C}}_{\mathrm{LZ}}\left(x\right),{\tilde{C}}_{\mathrm{LZ}}\left(y\right)\right\}$ under pickle+float32. Note the smooth uncorrelated case (**bottom-left**) can show appreciable $\rho_{\mathrm{obs}}$ purely from the shared random-walk structure—a spurious correlation. The $J_{\mathrm{LZ}}$ values saturate near 0.9 on Gaussian paths under pickle+float32 (Section 5.2), so the discrimination here is qualitative; the quantitative discrimination on Gaussian data comes from the pairwise false-positive curve (Figure 6b) rather than from the pointwise estimator.

Figure 7a shows the distribution of Pearson ρ for independent pairs at H=0.05 (rough) and H=0.90 (smooth). The smooth series distribution has heavy tails far beyond the threshold, while the rough series distribution is tightly concentrated near zero.

### 5.4. Bivariate mfBm: Genuine vs. Spurious Correlation

Figure 7b shows bivariate fBm paths under four combinations of roughness and target correlation $\rho_{\mathrm{target}}\in \left\{0,0.8\right\}$. The key distinction:

- Smooth (H=0.9) and uncorrelated ($\rho_{\mathrm{target}}=0$): both series drift similarly due to random walk behaviour—$\rho_{\mathrm{obs}}$ can be appreciably non-zero despite no true correlation, the Yule phenomenon.
- Rough (H=0.1) and correlated ($\rho_{\mathrm{target}}=0.8$): the observed correlation reflects genuine dependence because independent rough series would not align.

### 5.5. $J_{\;\mathrm{LZ}}$ as a Screening Criterion for mfBm Pairs

The joint complexity indicator $J_{\mathrm{LZ}}\left(x,y\right)=\min\left\{{\tilde{C}}_{\mathrm{LZ}}\left(x\right),{\tilde{C}}_{\mathrm{LZ}}\left(y\right)\right\}$ can be evaluated on any pair of fGn series and used as a pre-screening step before trusting a reported ρ. As observed in Section 5.2, however, ${\tilde{C}}_{\mathrm{LZ}}$ under this paper’s main serialisation saturates around 0.93 for all H∈[0.1,0.9], so $J_{\mathrm{LZ}}$ on a single pair of fGn series carries limited information in absolute terms. For stationary Gaussian-valued fGn under pickle+float32, pointwise ${\tilde{C}}_{\mathrm{LZ}}$ saturates and $J_{\mathrm{LZ}}$ is not an effective absolute threshold. In this regime, the useful calibration is instead the false-positive curve as a function of the memory/smoothness parameter *H* (or an estimated surrogate-family parameter). Thus, the fGn experiment calibrates the risk of Pearson false positives across a complexity-spanning family rather than providing a universal numerical cutoff on $J_{\mathrm{LZ}}$. The wider int8 dynamic range in Table 2 occurs on non-stationary fBm paths, not on fGn increments, and therefore should not be used as an increment-level screening threshold.

Where $J_{\mathrm{LZ}}$ does retain a useful screening role on mfBm data is the *non-stationary* case. fBm paths themselves (as opposed to their fGn increments) are non-stationary and produce very strong spurious correlations [48]; differing from the stationary fGn is Step 1 of the recipe in Section 7 and picked up automatically by the stationarity check in Remark 3. On the stationary fGn itself, the diagnostic power of the framework comes predominantly from the *pairwise* false-positive curve of Figure 6b—which is measured directly rather than inferred from individual complexities—and from the surrogate calibration.

**Remark 4.** 
*The operating threshold θ=0.3 used in $J_{\mathrm{LZ}}>\theta$ is a calibrated threshold for the bounded logistic examples and for data classes/encodings where $J_{\mathrm{LZ}}$ has useful dynamic range. It should not be read from the fGn complexity values. In the mfBm false-positive experiment, the left panel of Figure 6b gives a family-level risk calibration parameterised by H: the 5% transition occurs at H≈0.83, equivalently, for the associated fBm path graph, at $\dim_{H}\approx 1.17$. This transition cannot be re-expressed as an absolute ${\tilde{C}}_{\mathrm{LZ}}$ cutoff because, under pickle+float32, the pointwise ${\tilde{C}}_{\mathrm{LZ}}$ of fGn saturates near 0.93 across the entire Hurst range (Table 2), and coarsening to int8 does not change this for fGn increments. The pronounced int8 drop to ${\tilde{C}}_{\mathrm{LZ}}\approx 0.50$ is exhibited only by the non-stationary fBm paths (Table 2), which the stationarity pre-step removes before screening. The mfBm model therefore calibrates false-positive risk as a function of the memory/smoothness family, not a universal numerical cutoff on $J_{\mathrm{LZ}}$.*

### 5.6. Summary of Experiment 2

The mfBm experiment complements Experiment 1 by grounding this paper’s framework in continuous stochastic processes whose fBm path graphs have an analytically known Hausdorff dimension. Three main findings emerge.

#### 5.6.1. Complexity and Hausdorff Dimension Are Empirically Linked

The Hurst parameter *H* controls the Hausdorff dimension of the associated fBm path exactly as $\dim_{H}=2-H$. Figure 6a shows that this path-level theoretical quantity is tracked qualitatively—monotonically but not strongly—by ${\tilde{C}}_{\mathrm{LZ}}$ under this paper’s pickle+float32 serialisation: ${\tilde{C}}_{\mathrm{LZ}}$ on fBm paths varies only between ≈0.92 at H=0.1 and ≈0.90 at H=0.9, a ∼2% relative trend over the full roughness range. The same data under int8 quantisation produce a much sharper trend (${\tilde{C}}_{\mathrm{LZ}}\approx 0.95$ at H=0.1 down to ≈0.50 at H=0.9; Table 2). Spectral entropy $\tilde{H}$ tracks this path-level dimension more strongly under all schemes. The pointwise estimator is therefore consistent with the Section 2 picture qualitatively but is a weak quantitative proxy for $\dim_{H}$ on Gaussian-valued data; the operationally meaningful signal is the pairwise false-positive rate of the next paragraph.

#### 5.6.2. False-Positive Rate Is Controlled by the Memory/Smoothness Parameter *H*

The key result (Figure 6b) shows that spurious correlations among independent fGn pairs are suppressed as the memory/smoothness parameter *H* decreases (equivalently, as the associated fBm paths become rougher and have larger graph dimension); under pickle+float32, pointwise LZ complexity only weakly reflects this transition because ${\tilde{C}}_{\mathrm{LZ}}$ saturates. The transition is sharp and monotone: pairs with H≲0.80 have FP rates ≤1.8%, while pairs at H=0.85 and H=0.90 produce spurious correlations in 7.2% and 20.8% of trials, respectively. The false-positive rate is the primary quantity of interest here; it is measured directly as a function of *H* in Figure 6b and drives the family-level risk calibration in Section 7. The centre panel of Figure 6b, which expresses the same rate against ${\tilde{C}}_{\mathrm{LZ}}$, is a post hoc reparameterisation; because ${\tilde{C}}_{\mathrm{LZ}}$ saturates for fGn (Section 5.2), this curve should be read as showing the false-positive transition as *H* crosses into the very smooth regime, rather than as a monotone complexity-dependent law.

#### 5.6.3. Non-Stationarity Is a Separate Confound That Complexity Alone Cannot Resolve

The fBm paths (as opposed to their fGn increments) show uniformly high false-positive rates regardless of *H*, replicating the classical Yule nonsense-correlation phenomenon. The appropriate response is to difference first (recovering the stationary fGn) and then apply the complexity screen. This two-stage pipeline (stationarity test → complexity screen) is the recommended practical procedure.

#### 5.6.4. $J_{\mathrm{LZ}}$ as a Screening Criterion on mfBm

On stationary fGn, $J_{\mathrm{LZ}}$ does not function as an absolute or rank-based cutoff: as noted above, ${\tilde{C}}_{\mathrm{LZ}}$ saturates for Gaussian-valued increments under pickle+float32, so $J_{\mathrm{LZ}}$ on a single fGn pair carries little information. What the fGn experiment calibrates is the risk of Pearson false positives as a function of the memory/smoothness parameter *H* across a complexity-spanning family. The mfBm family therefore provides a natural calibration environment for screening risk, even though the absolute ${\tilde{C}}_{\mathrm{LZ}}$ scale carries limited information on its own.

#### 5.6.5. Direction of the Random-Shuffle Comparison

Random-shuffle surrogates destroy temporal dependence while preserving the one-point marginal distribution. Therefore, a smooth or strongly autocorrelated series should typically have *lower* compression complexity than its shuffled surrogate because the shuffle removes the temporal regularity and produces a more noise-like (less compressible) sequence. Conversely, a rough or nearly noise-like series may already lie close to its shuffled surrogate in compression complexity. Shuffle surrogates are thus useful for detecting temporal structure, but they should not be confused with the independent matched-memory surrogates used to estimate false-positive correlation rates (Section 7); the two answer different questions.

## 6. Comparison with Existing Spurious Correlation Diagnostics

A natural question is how the complexity-filtered correlation test relates to existing methods for detecting spurious correlations. We briefly discuss four alternatives and clarify the complementary role of our approach. Table 6 summarises the contrast in one place.

The proposed $J_{\mathrm{LZ}}$ screen is meant to complement, not replace, the methods above. We now discuss each in turn.

### 6.1. Permutation (Surrogate) Testing

The standard approach to testing whether an observed ρ is statistically significant is to generate surrogate pairs by randomly permuting one series, compute ρ for each surrogate pair, and reject the null hypothesis of independence if the observed ρ exceeds the *p*-th quantile of the surrogate distribution [8]. For i.i.d. data, this controls the false-positive rate at any desired level. However, for time series with temporal structure (e.g., autocorrelation, periodicity, or long-range dependence), naïve permutation destroys the temporal dependence and produces anti-conservative *p*-values. Phase-randomisation surrogates [8] preserve the power spectrum but not higher-order structure.

Common surrogate-generation methods include random-shuffle surrogates for testing independence under an i.i.d. null, Fourier-transform surrogates that preserve the power spectrum while randomising the phase, and amplitude-adjusted Fourier surrogates that additionally preserve the amplitude distribution of the original signal [43]. These approaches test specific stochastic null models, whereas the complexity diagnostic proposed here instead evaluates the structural richness of the time series themselves.

It is important to recognise that surrogate testing and complexity filtering address *fundamentally different null hypotheses* and are therefore complementary:

- **Surrogate testing** asks: “Is the observed correlation consistent with the data being i.i.d. or linear noise?” A significant result means the correlation exceeds what would arise from the chosen null model.
- **Complexity filtering** asks: “Given the structural richness (complexity) of the series involved, how surprising is the observed level of correlation?”

A correlation can be “significant” against a red-noise null (surrogates reject) yet still be spurious if both series are simple monotone trends. This is precisely the class of cases highlighted by the Tyler Vigen examples (Figure 1). We recommend using both tools in tandem: surrogate testing to establish that the correlation exceeds what would arise from linear stochastic processes, and $J_{\mathrm{LZ}}$ to assess whether the data are complex enough for that correlation to be informative.

### 6.2. Cointegration and Unit-Root Tests

For non-stationary series, the Augmented Dickey–Fuller test and Johansen cointegration test [9] are standard tools. These directly address the Yule phenomenon (integrated series spuriously correlate). Our recommendation to first difference or detrend non-stationary series before applying the complexity diagnostic (Section 9, Remark 3) is fully consistent with this classical approach. The complexity diagnostic adds value *after* stationarity is established by distinguishing genuinely complex stationary dynamics from simple periodic or near-periodic patterns that can also produce spurious alignment.

### 6.3. MDL-Based Causal Inference

Methods like GLOBE [10] and CASCADE [11] use the Minimum Description Length principle—a computable approximation to Kolmogorov complexity—to infer full causal DAGs. Our $J_{\mathrm{LZ}}$ test is a lightweight *screening* step that can be applied before expensive causal discovery: pairs failing the complexity threshold (low $J_{\mathrm{LZ}}$) are flagged as likely spurious without running a full MDL search.

### 6.4. Normalised Compression Distance (NCD)

A natural candidate baseline is the *Normalised Compression Distance* of Cilibrasi and Vitányi [23,24], a compression-based approximation to the Universal Similarity Metric:

$$\mathrm{NCD}\left(x,y\right)\;=\;\frac{C(xy)-\min\{C(x),C(y)\}}{\max\{C(x),C(y)\}}\;\in \left[0,1\right],$$

where C(·) denotes the compressed length under a lossless compressor (we use the same zlib used for ${\tilde{C}}_{\mathrm{LZ}}$). Theoretically, NCD→0 iff x and y are maximally algorithmically dependent (one a computable function of the other), and NCD→1 iff they are algorithmically independent.

It is important to recognise that NCD and $J_{\mathrm{LZ}}$ answer *different* questions:

- NCD asks: *are x and y algorithmically dependent?* (Does one help compress the other?)
- $J_{\mathrm{LZ}}$ asks: *are x and y each individually complex enough that an observed Pearson correlation is unlikely to be accidental?*

They are therefore complementary rather than competing: NCD is a dependence detector, while $J_{\mathrm{LZ}}$ is a reliability filter on Pearson correlations. A thorough workflow could use both in sequence: $J_{\mathrm{LZ}}$ to decide whether a reported correlation is worth investigating, then NCD to confirm algorithmic dependence directly.

### 6.5. Empirical Comparison on Coupled Logistic Maps

**Figure 8:**
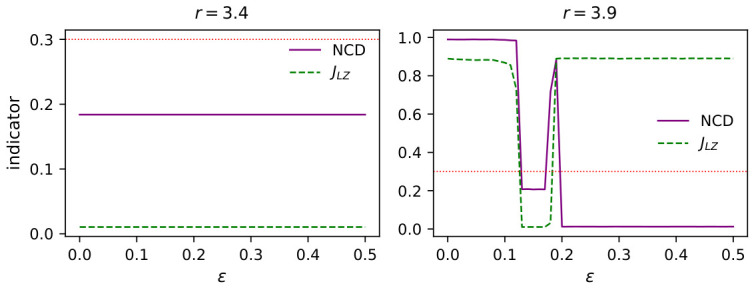
NCD (purple solid) and $J_{\mathrm{LZ}}$ (green dashed) as functions of coupling ε for coupled logistic maps. (**Left**) r=3.4 (periodic). (**Right**) r=3.9 (chaotic). The red dotted line marks the screening threshold θ=0.3 on $J_{\mathrm{LZ}}$. NCD detects the *algorithmic coupling* of the two series (drops from ≈0.99 at ε=0 to ≈0.01 once $\varepsilon \geq \varepsilon_{c}\approx 0.19$ in the chaotic regime), while $J_{\mathrm{LZ}}$ detects *individual complexity* (stays at ≈0.89 throughout the chaotic regime apart from a narrow pre-synchronisation collapse window ε∈[0.13,0.18] and stays at ≈0.01 throughout the periodic regime). The two indicators are complementary rather than competing: NCD answers “are these series joint-compressible?”; $J_{\mathrm{LZ}}$ answers “are these series individually complex?”.

Figure 8 shows NCD alongside $J_{\mathrm{LZ}}$ and Pearson ρ on the coupled logistic system for both regimes. The two complexity-based indicators behave qualitatively differently:

- In the chaotic regime (r=3.9), as coupling increases from ε=0 to ε=0.5, NCD drops monotonically from ≈0.99 (algorithmically independent) at ε=0 to ≈0.01 (maximally dependent) once $\varepsilon \geq \varepsilon_{c}$, correctly detecting the synchronisation transition. Over the same sweep, $J_{\mathrm{LZ}}$ stays at ≈0.89 outside the narrow pre-synchronisation window ε∈[0.13,0.18], where it momentarily collapses to ≈0.01 together with the individual complexities (the symmetric joint-collapse phenomenon of Section 4); for ε≥0.20, it returns to ≈0.89 because both series continue to trace high-complexity chaotic orbits, just the *same* orbit after synchronisation. Both indicators thus carry information, but about different physical quantities: NCD encodes pairwise dependence; $J_{\mathrm{LZ}}$ encodes pointwise complexity. They agree on the screen verdict (“trustworthy” on both sides of the transition window) but disagree on the dependence structure (NCD low after sync; $J_{\mathrm{LZ}}$ blind to coupling per se).
- In the periodic regime (r=3.4), NCD≈0.28 across all ε (partial dependence from the shared period-2 structure) while $J_{\mathrm{LZ}}\approx 0.01$ across all ε (both series are simple and compressible; cf. the pickle+float32 periodic value ${\tilde{C}}_{\mathrm{LZ}}\approx 0.011$ in Table 2).

Both behaviours are correct, and neither indicator can substitute for the other: $J_{\mathrm{LZ}}$ cannot tell us that synchronised chaotic maps are dependent (they both look complex individually), while NCD cannot tell us that the spurious Pearson ρ=1 in the periodic regime is coming from simple, near-identical orbits rather than a genuine coupling signal.

### 6.6. When $J_{\mathrm{LZ}}$ Is Preferable

For the specific task of screening a reported Pearson correlation—“is this number worth trusting?”—$J_{\mathrm{LZ}}$ has three practical advantages: *(i) Computational cost.* $J_{\mathrm{LZ}}$ is structurally cheaper for screening tasks. For a population of *K* candidate series, $J_{\mathrm{LZ}}$ requires only *K* individual compressions (each O(N) with zlib’s bounded-window DEFLATE) since the indicator depends on the two marginal complexities and these can be cached per series and reused across all K(K−1)/2 pairs. NCD, by contrast, requires $O(K^{2})$ pairwise compressions of the concatenated series xy at O(N) each since the joint compressed length cannot be obtained from the marginals. The asymptotic gap is therefore O(K) vs. $O(K^{2})$ in compressions; for K=1000, this is roughly three orders of magnitude. *(ii) Calibration:* the threshold or family rule for $J_{\mathrm{LZ}}$ can be calibrated using independent surrogate or complexity-spanning calibration ensembles (Section 7); NCD lacks an equally clean calibration. *(iii) Design intent:* $J_{\mathrm{LZ}}$ is designed to *complement* Pearson correlation, whereas NCD replaces it with a different similarity measure altogether.

### 6.7. When NCD Is Preferable

Conversely, NCD has genuine advantages: *(i) Metric structure:* NCD is (approximately) a metric on the space of sequences, with strong information-theoretic guarantees [24]; $J_{\mathrm{LZ}}$ is a heuristic screening indicator. *(ii) Direct dependence detection:* if the question of interest is “are these two sequences algorithmically related?” (e.g., phylogenetic inference, plagiarism detection), NCD answers it directly, whereas $J_{\mathrm{LZ}}$ only rules out trivial spuriousness. *(iii) Fine-grained similarity:* in the intermediate regime 0<NCD<1, it gives a continuous similarity score; $J_{\mathrm{LZ}}$ gives only a pass/fail decision at the chosen threshold.

## 7. Threshold Selection and Sensitivity

The false-positive rate thresholds (|ρ|>0.3 for logistic maps, |ρ|>0.1 for fGn) and the $J_{\mathrm{LZ}}$ threshold of 0.3 deserve justification.

For the correlation thresholds, we chose |ρ|>0.3 in the logistic experiment because the periodic regime (r=3.4) produces deterministic orbits where ρ∈{−1,+1} exactly, so any reasonable threshold yields the same qualitative result. The fGn experiment uses the stricter |ρ|>0.1 because the effect is subtler for stationary Gaussian processes; we verified that the monotone relationship between false-positive rate and Hausdorff dimension persists for thresholds 0.05,0.10,0.15, and 0.20 (the curves shift vertically but the ordering is unchanged).

For the $J_{\mathrm{LZ}}$ threshold, the value 0.3 is motivated by a two-step argument. First, Table 2 shows that under pickle+float32 serialisation, a truly periodic logistic trajectory has ${\tilde{C}}_{\mathrm{LZ}}\approx 0.011$, so the noise floor in the idealised periodic case is effectively zero. The practical floor is set instead by finite-sample effects, small departures from pure periodicity, and (analytically) the Posobin–Shen contribution H(τ) from measurement noise: for typical noise rates τ∈[0.01,0.05], the analytical floor sweeps the interval [0.08,0.29] (Section 2.6), with the upper end close to θ=0.3. For N=5000, we observe ${\tilde{C}}_{\mathrm{LZ}}≲0.1$ for smooth logistic signals (periodic windows, damped oscillations) in the noiseless case; the screening threshold must be strictly above this floor and should be re-calibrated upward when measurement noise is substantial. Second, the fGn false-positive experiment (Figure 6b, *left* panel) provides family-level calibration for the smooth-process regime: the false-positive rate drops below 5% as *H* falls below ≈0.83. This transition is parameterised by *H* rather than by an absolute ${\tilde{C}}_{\mathrm{LZ}}$ value: under pickle+float32, the pointwise ${\tilde{C}}_{\mathrm{LZ}}$ of fGn saturates near 0.93 for all *H*, and even under int8 it remains near 0.945 for all *H* (Table 2), so the transition is invisible in ${\tilde{C}}_{\mathrm{LZ}}$ space under either serialisation. Calibration must therefore be performed by estimating false-positive risk across a complexity-spanning family parameterised by *H* (or by an estimated memory/smoothness parameter), rather than by ranking $J_{\mathrm{LZ}}$ against a matched-memory null or by applying a fixed ${\tilde{C}}_{\mathrm{LZ}}$ threshold. The value θ=0.3 is retained as a conservative operating point for the bounded logistic pipeline; for fGn-type Gaussian-valued processes, the pointwise ${\tilde{C}}_{\mathrm{LZ}}$ under pickle+float32 sits near 0.93 for all *H* (Table 2), so risk calibration must be parameter-family-based rather than an absolute or rank-based $J_{\mathrm{LZ}}$ test.

### 7.1. Do Not Transfer fBm-Path Thresholds to fGn Increments

It is important not to confuse thresholds calibrated on fBm *paths* with thresholds calibrated on fGn *increments*. The stationarity-first workflow recommends applying the complexity screen after differencing non-stationary paths, when differencing is appropriate; therefore, if the analysed object is the stationary increment process, θ must be calibrated on fGn-like calibration ensembles, not on the original fBm paths. In particular, the int8 compression transition observed for smooth fBm paths should not be used as an absolute threshold for fGn increments: for fGn under the encodings considered here, pointwise ${\tilde{C}}_{\mathrm{LZ}}$ may remain near saturation across *H*, so the false-positive transition is better captured by the pairwise correlation false-positive curve (Figure 6b) than by a universal pointwise compression threshold. We therefore do *not* recommend a universal value θ=0.3 for all Gaussian processes and all encodings: θ=0.3 should be understood as a toy-model threshold for the documented serialisation and calibration ensemble. For stationary Gaussian increments, threshold selection must be repeated on a calibration family that spans the relevant smoothness or memory parameter.

### 7.2. Practical Recipe for Threshold Selection

We consolidate the threshold discussion into the following step-by-step procedure:

1. **Compute**$J_{\mathrm{LZ}}$ for the pair of series under consideration.
2. **Establish a noise floor.** Under pickle+float32 serialisation, a truly periodic logistic orbit gives ${\tilde{C}}_{\mathrm{LZ}}\approx 0.011$; smooth but non-periodic dynamics typically give ${\tilde{C}}_{\mathrm{LZ}}≲0.1$ (for N=5000). For Gaussian-valued processes such as fGn, ${\tilde{C}}_{\mathrm{LZ}}$ saturates near 0.93 for all Hurst parameters under pickle+float32 (near 0.96 under struct+float64), so the threshold discussion proceeds via surrogate calibration (Step 3) rather than against an absolute floor.
3. **Generate surrogate and calibration distributions** for independent pairs under the null hypothesis of independence. Two *distinct* purposes must be kept separate here: *local surrogate assessment* (is this correlation surprising within this marginal class?) and *global threshold calibration* (which complexity threshold to use across classes?).
  (a) *Local surrogate assessment (matched-marginal independent surrogates).* Given the observed pair (x,y), generate independent surrogates $\left(x^{\left(b\right)},y^{\left(b\right)}\right)$ that preserve the relevant marginal features of x and y—length, marginal distribution, variance, and, when appropriate, the memory or smoothness class—and compute ρ on them. This estimates the probability that a correlation as large as the observed one could arise under independence *within the same marginal class*. It is the most general construction and works for any data class. Note, however, that if the surrogates are matched in smoothness, marginal distribution, and memory, their $J_{\mathrm{LZ}}$ values may be nearly constant, so this construction alone cannot select a global threshold.
  (b) *Global threshold or family calibration (complexity-spanning ensemble).* A global screening rule cannot be calibrated from a single matched-marginal surrogate class if that class has nearly constant $J_{\mathrm{LZ}}$. For signal classes where $J_{\mathrm{LZ}}$ has meaningful dynamic range, one needs a calibration ensemble that *spans* a range of complexities—for example an *H*-sweep of fGn or fBm surrogates, a parameter sweep over dynamical regimes, or a mixture of representative null models. Such a family allows one to estimate $\mathrm{Pr}(|\rho |>\tau |J_{\mathrm{LZ}}>\theta )$ as a function of θ. If the Hurst exponent of stationary Gaussian-increment data is approximately known (or estimable from the spectral slope), the false-positive risk should instead be read from the mfBm curve as a function of *H* (Figure 6b, left panel).
  In short: matched surrogates answer “Is this correlation surprising within this marginal class?”; they do not, by themselves, answer “Which global complexity threshold should be used across classes?”. We also deliberately do *not* recommend permuting one series and recomputing $J_{\mathrm{LZ}}$ on the permuted pair: this construction is uninformative for the min-based indicator, since $J_{\min}\left(x,\pi \left(y\right)\right)=\min\left({\tilde{C}}_{\mathrm{LZ}}\left(x\right),{\tilde{C}}_{\mathrm{LZ}}\left(\pi \left(y\right)\right)\right)$ collapses to ${\tilde{C}}_{\mathrm{LZ}}\left(x\right)$ as ${\tilde{C}}_{\mathrm{LZ}}\left(\pi \left(y\right)\right)\to 1$ under permutation, and the observed $J_{\mathrm{LZ}}\left(x,y\right)$ is by construction always $\leq {\tilde{C}}_{\mathrm{LZ}}\left(x\right)$, making the comparison one-sided and trivial.
4. **Set the threshold or family rule.** For signal classes and encodings where $J_{\mathrm{LZ}}$ has meaningful dynamic range, calibrate θ against the conditional false-positive rate of ρ as a function of the screening indicator: evaluate $\mathrm{Pr}(|\rho |>\tau |J_{\mathrm{LZ}}>\theta )$ across the complexity-spanning calibration population of Step 3 and choose the smallest θ at which this conditional rate falls below the desired significance level α (e.g., α=0.05). The value θ=0.3 is calibrated and valid for the bounded logistic examples (α=0.05). For stationary Gaussian increments under fine serialisation, however, $J_{\mathrm{LZ}}$ saturates; the mfBm curve of Figure 6b should therefore be read as a false-positive calibration as a function of *H* or the relevant memory/smoothness parameter, not as an absolute $J_{\mathrm{LZ}}$ cutoff.
5. **Screen:** if the data class has a calibrated $J_{\mathrm{LZ}}$ threshold and $J_{\mathrm{LZ}}>\theta$, and |ρ| is large, the correlation is “complexity-supported” and warrants further investigation. If $J_{\mathrm{LZ}}<\theta$, flag the correlation as potentially spurious regardless of |ρ|. For saturating Gaussian-increment data, use the estimated memory/smoothness family and false-positive curve instead of an absolute or rank-based $J_{\mathrm{LZ}}$ cutoff.

**Figure 9:**
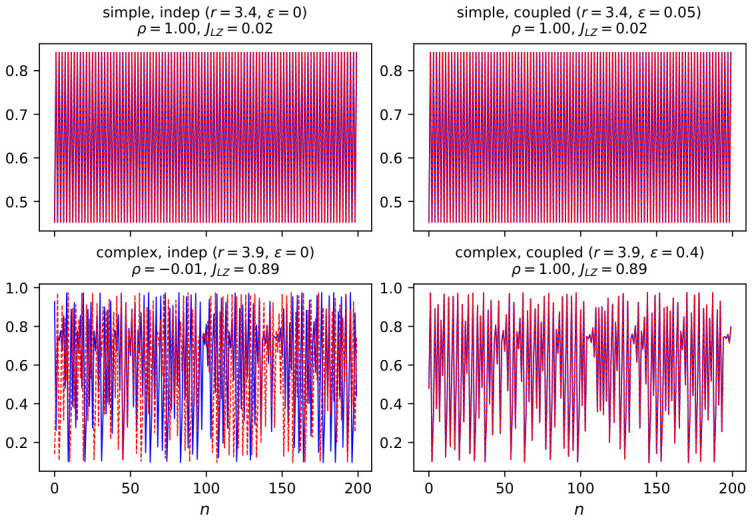
The four regimes of (complexity, correlation), illustrated on the coupled logistic map. Annotated values of ρ and $J_{\mathrm{LZ}}=\min\left\{{\tilde{C}}_{\mathrm{LZ}}\left(x\right),{\tilde{C}}_{\mathrm{LZ}}\left(y\right)\right\}$ are empirical (pickle+float32 serialisation, N=2000). (**Top-left**) (simple, independent ICs, r=3.4, ε=0): two independent period-2 orbits phase-lock in phase in steady state, so ρ≈+1 despite zero coupling—|ρ|≈1 regardless, a clean illustration of why classical ρ is uninformative for simple systems. $J_{\mathrm{LZ}}\approx 0.01$ correctly flags the pair as untrustworthy. (**Top-right**) (simple, weakly coupled): same attractor, ρ≈+1, same conclusion. (**Bottom-left**) (complex, independent, r=3.9, ε=0): two chaotic trajectories are visibly uncorrelated (ρ≈0) and $J_{\mathrm{LZ}}\approx 0.89$ confirms joint complexity. (**Bottom-right**) (complex, strongly coupled ε=0.4): synchronised chaotic trajectories with $x_{n}=y_{n}$, so ρ≈+1, and $J_{\mathrm{LZ}}\approx 0.89$ indicates that the correlation is supported because both series individually remain complex. $J_{\mathrm{LZ}}$ correctly assigns high scores only to the two bottom cases; the two top cases are screened out regardless of ρ.

The quadrant diagram (Figure 9) provides the primary interpretive tool; the numerical threshold is secondary and should be adapted to the application domain.

Figure 9 illustrates this paper’s central message with concrete examples from the logistic map in all four complexity-correlation regimes.

## 8. Illustration on Stylised Real-World Patterns

While our main results are demonstrated on controlled toy models (where ground truth is known), we briefly illustrate how the complexity diagnostic applies to real-world-like patterns.

### 8.1. Monotone Trends (Tyler Vigen Class)

The canonical spurious correlations of Figure 1 involve nearly monotone time series (e.g., the distance between Neptune and the Sun, or the popularity of a name over time). Such series have very low LZ complexity (${\tilde{C}}_{\mathrm{LZ}}\approx 0.02$–0.08) because a monotone trend of length *N* can be encoded by its start, end, and a short polynomial description. Any pair of such series trivially lands in the “low $J_{\mathrm{LZ}}$, high |ρ|” quadrant—precisely the regime our framework flags as likely spurious. This is what practitioners may already suspect intuitively; the contribution of our framework is to make this intuition quantitative and to connect it to Kolmogorov complexity and the Hausdorff dimension of the underlying attractor.

### 8.2. Financial Return Series

As a contrasting example, consider daily log-returns of two stock indices (e.g., S&P 500 and FTSE 100). These series are approximately i.i.d. with heavy tails—they have high LZ complexity (${\tilde{C}}_{\mathrm{LZ}}\approx 0.85$–0.92) and high spectral entropy ($\tilde{H}\approx 0.90$–0.95). When two such series exhibit a moderately high correlation (ρ≈0.6), this lands in the “high $J_{\mathrm{LZ}}$, high |ρ|” quadrant and should be taken seriously as evidence of genuine co-movement—consistent with the well-known common-factor structure of global equity markets. We emphasise that our framework does not *prove* causation; it merely identifies which correlations are worth investigating further.

### 8.3. Limitations of the Real-World Illustration

We deliberately refrain from a full empirical study on real data because the ground truth (genuine vs. spurious) is typically unknown. Our toy models provide the controlled setting needed to validate the theoretical claims. Extending the framework to large-scale empirical benchmarks (e.g., the CauseMe platform [5] or financial datasets with known factor structure) is an important direction for future work.

## 9. Discussion and Practical Recommendations

### 9.1. Summary

We have demonstrated, through a theoretical framework grounded in algorithmic information theory and empirical validation on two toy models, that spurious correlations are far more prevalent among low-complexity (simple, smooth) time series than high-complexity (chaotic, rough) ones (Cf. [32]). The theoretical argument assembles three known results into a novel narrative for time-series analysis: (1) the Solomonoff prior assigns higher probability to simple patterns [16]; (2) the Lutz–Mayordomo theorem [33] equates normalised Kolmogorov complexity with the effective Hausdorff dimension; (3) the Posobin-Shen result [40] quantifies how noise inflates observed complexity. We invoked these known results to address the problem of spurious correlations arising from the occurrence of simple patterns in data. We presented a method of how to measure complexity of pairs of series via the $J_{\mathrm{LZ}}=\min\left\{{\tilde{C}}_{\mathrm{LZ}}\left(x\right),{\tilde{C}}_{\mathrm{LZ}}\left(y\right)\right\}$ and studied examples numerically to investigate how our framework works in practice. Beyond confirming the main thesis, the experiments revealed an additional finding: in the chaotic logistic map, $J_{\mathrm{LZ}}$ detects the pre-synchronisation collapse of individual complexity near $\varepsilon_{c}$, a genuine dynamical event invisible to similarity-based indicators such as $S_{\mathrm{LZ}}$.

### 9.2. Noise Floor

The Posobin–Shen result implies that real observed series have their LZ complexity inflated by measurement noise. The binary Shannon entropy is H(0.01)≈0.081, H(0.02)≈0.141, H(0.05)≈0.286, so for typical noise rates τ∈[0.01,0.05], the analytical lower bound on the complexity rate sweeps the interval [0.08,0.29]. The upper end of this range sits close to the bounded-logistic operating threshold θ=0.3, so applications with appreciable measurement noise should re-calibrate the screening rule against a noise-matched ensemble. The corresponding empirical noise floor on ${\tilde{C}}_{\mathrm{LZ}}$ under pickle+float32 is much lower in absolute terms: a truly periodic logistic orbit gives ${\tilde{C}}_{\mathrm{LZ}}\approx 0.011$ (Table 2), and smooth-but-not-periodic dynamics give ${\tilde{C}}_{\mathrm{LZ}}≲0.1$ at N=5000. The operating value θ=0.3 for $J_{\mathrm{LZ}}$ is calibrated empirically for the bounded logistic examples rather than analytically from a bit-count argument. The fGn experiment supplies a family-level false-positive calibration as a function of memory/smoothness; practitioners applying the framework to a different signal class should re-calibrate the screening rule against a data-class-appropriate calibration ensemble.

### 9.3. Practical Recommendation

When reporting correlations between time series, we recommend:

1. Report ${\tilde{C}}_{\mathrm{LZ}}$ for each series alongside ρ.
2. Use $J_{\mathrm{LZ}}$ as the joint complexity indicator.
3. Apply extra skepticism when $J_{\mathrm{LZ}}<0.3$ in a data class where this threshold has been calibrated (both series are simple or of very different complexity); for stationary Gaussian increments, use the estimated memory/smoothness family and the false-positive calibration curve instead of a universal $J_{\mathrm{LZ}}$ cutoff.
4. For non-stationary series, complexity alone is insufficient, so first-difference or detrend before analysis (Remark 3).
5. Use this diagnostic as a complement to, not a replacement for, standard significance testing (Section 6).

### 9.4. Limitations

We summarise the principal scope restrictions of the framework as follows:

1. The formal theory applies to finite binary strings (Propositions 1 and 2) and to infinite binary sequences (Propositions 3 and 4) under Hamming correlation, not directly to arbitrary real-valued time series under Pearson correlation.
2. The effective-Hausdorff-dimension bridge of Section 2.5.1 justifies the Hausdorff-dimension framing of the binary-sequence theory but does *not* prove that LZ compression of a serialised real-valued time series is a consistent estimator of Kolmogorov complexity, effective Hausdorff dimension, or classical Hausdorff dimension.
3. Compression ratios depend on the serialisation scheme, quantisation, compressor, and  sample length (Table 2); $J_{\mathrm{LZ}}$ is therefore not invariant across encodings, and thresholds must be re-calibrated for each application.
4. On stationary Gaussian-increment processes such as fGn under fine serialisation, ${\tilde{C}}_{\mathrm{LZ}}$ may saturate across the relevant memory range, so $J_{\mathrm{LZ}}$ should not be used as an absolute or rank-based cutoff; calibration should instead be expressed through the estimated memory/smoothness family.
5. Non-stationary stochastic processes (e.g., random walks, integrated processes) can simultaneously be algorithmically complex and produce spuriously high Pearson correlations; the stationarity pre-step is essential and is not provided by the complexity screen itself.
6. $J_{\mathrm{LZ}}$ is a theoretically motivated, empirically calibrated diagnostic and not a causal test; passing the screen identifies correlations worth investigating, not causal relationships.
7. Thresholds must be calibrated against a null model appropriate to the data class (Section 7).
8. Short time series (N<500) may give unreliable LZ estimates due to compressor dictionary overhead.
9. The empirical relationship between the chosen finite-resolution encoding and any classical Hausdorff-dimension claim is indirect, and quantitative claims of the form ${\tilde{C}}_{\mathrm{LZ}}\approx 2-H$ should be interpreted as resolution-dependent empirical phenomena rather than as identities.

We now elaborate on the most consequential of these limitations. First, our toy models each have a single parameter controlling complexity (the logistic parameter *r* or the Hurst exponent *H*); real time series exhibit mixed dynamics, seasonality, structural breaks, and multiple confounders. To partially address this, we tested a “mixed-complexity” signal $x\left(t\right)=A\sin\left(2\pi f_{0}t\right)+\eta \left(t\right)$, where η(t) is fractional Gaussian noise with Hurst exponent *H*. By varying the amplitude ratio $A/\sigma_{\eta }$, we sweep from a regime dominated by the simple periodic component (low complexity) to one dominated by the complex stochastic component (high complexity). Forming pairs of such mixed signals with independent noise realisations and measuring the false-positive rate, we find that $J_{\mathrm{LZ}}$ correctly tracks the effective complexity of the mixture: when the periodic component dominates ($A/\sigma_{\eta }\gg 1$), $J_{\mathrm{LZ}}$ is low and false positives are frequent; as the noise component dominates, $J_{\mathrm{LZ}}$ rises and false positives drop, consistent with the theory.

In practice, common real-world complications should be handled as follows: (a) for multiregime series, one should consider windowed complexity estimates; (b) seasonal and trend components should be removed prior to complexity estimation (analogous to the stationarity requirement of Remark 3); and (c) structural breaks locally inflate observed complexity, which may actually be desirable to detect. The complexity diagnostic is meant to be used alongside standard preprocessing (detrending, differencing) rather than as a standalone tool.

Second, the LZ complexity estimator depends on the serialisation scheme and series length (see Section 3), and its relationship to the true Kolmogorov complexity is only asymptotic. Third, the $J_{\mathrm{LZ}}$ indicator treats both series symmetrically. For asymmetric pairs (one complex, one simple, e.g., ${\tilde{C}}_{\mathrm{LZ}}\left(x\right)=0.8$, ${\tilde{C}}_{\mathrm{LZ}}\left(y\right)=0.2$), the min-based primary indicator gives $J_{\mathrm{LZ}}=0.2$, which lies below the threshold θ=0.3 and correctly flags the pair as untrustworthy: the smoother series is the weak link and dominates the score. Practitioners preferring a softer penalty on asymmetry may substitute the geometric-mean alternative $J_{\mathrm{geom}}=\sqrt{{\tilde{C}}_{\mathrm{LZ}}\left(x\right){\tilde{C}}_{\mathrm{LZ}}\left(y\right)}$, which would score this pair at $\sqrt{0.16}=0.40$; the two indicators agree to four decimal places on symmetric data.

A specific class of asymmetric relationships deserves explicit comment: the case of a slow exogenous *driver* (low complexity) entraining a fast complex *response* (high complexity)—e.g., a 24 h solar forcing entraining a chaotic biological rhythm. Here, the two series are genuinely related, but $J_{\min}$ flags them as untrustworthy because the driver itself is simple. This is by design: the screen exists to suppress false positives among low-complexity data, and the cost of doing so is precisely such asymmetric false negatives. The operationally honest answer is to report *both* $J_{\min}$ (conservative gate) and $J_{\mathrm{geom}}$ (softer alternative) alongside ρ and to escalate confirmed asymmetric candidates to a full causal-discovery pipeline (GLOBE [10], CASCADE [11]) rather than to redesign the screen around the driver–response case. Detecting genuine asymmetric causation is a different task from filtering spurious symmetric correlations, and the right tool for it is a directional dependence estimator, not a complexity floor.

Fourth, the LZ-based pipeline requires N≥500 for stable ${\tilde{C}}_{\mathrm{LZ}}$ estimates; below this length, the compressor’s dictionary overhead dominates. For short-panel applications (N∼100–500), two computable alternatives carry the same information at better small-sample behaviour: the spectral entropy $\tilde{H}\left(x\right)$ already used in this paper as a Fourier-domain proxy, and the permutation entropy of Bandt and Pompe [49], whose finite-sample bias is well characterised. The algorithmic-trilemma argument of Section 2.4 suggests that any proxy monotone in entropy rate can support the same screening principle, but the resulting threshold must be calibrated empirically for the chosen proxy and data class. Adapting the surrogate-calibration recipe of Section 7 to short-panel proxies is left as future work; we have not run the empirical experiments at N<500 in the present paper because the mfBm calibration curve we rely on is itself measured at N=2000.

Fifth, the spectral entropy measure $\tilde{H}$ is only one possible Fourier-domain proxy; wavelet-based alternatives may be more appropriate for non-stationary signals.

### 9.5. Connections and Future Work

Our complexity-filtered correlation test is a lightweight *screening* step that naturally precedes full causal discovery methods such as GLOBE [10] and CASCADE [11], which use MDL/Kolmogorov complexity principles to recover directed acyclic graphs. Pairs passing our test (high $J_{\mathrm{LZ}}$ and high ρ) are natural candidates for deeper causal analysis; we emphasise, however, that our framework identifies correlations *worth investigating*, not causal relationships per se.

The multivariate fBm model [45] provides a rich testbed for further experiments: varying $H_{1}\neq H_{2}$ (series of mismatched roughness), exploring the asymmetry parameter $\eta_{ij}$, and testing on the long-range dependent regime H>0.5 where spurious correlations in the non-stationary case are known to be especially severe [48]. Validating the framework on real-world benchmarks with known causal structure (e.g., CauseMe [5]) and developing sample-size corrections for ${\tilde{C}}_{\mathrm{LZ}}$ in the short-series regime (N<200) are important next steps. A further open direction, posed explicitly in Section “An Open Question: Task-Optimal Serialisation”, is to formalise the task-optimal serialisation: given a signal class and discrimination task, what quantisation maximises the task-relevant mutual information at fixed description length? This would replace the empirical choice documented in Table 2 with a principled one. The connection to algorithmic mutual information I(x:y)=K(x)+K(y)−K(x,y) (Section 2.7) also deserves further development: an *empirical* joint-complexity measure based on compressing the concatenated series (x,y) could provide a direct estimator of I(x:y) and offer a richer diagnostic than $J_{\mathrm{LZ}}$ alone.

Our work contributes to ongoing developments in the field of Kolmogorov complexity applications to dynamical series and time series [50,51,52,53,54,55,56,57].

### 9.6. Relation to a Broader Programme

The complexity-based perspective developed here is consistent with, and complements, an ongoing programme bridging algorithmic information theory and machine learning [58,59,60,61,62,63,64,65,66,67,68,69,70,71], whose foundational parts appeared in *Physica D* [58,59]. That programme develops Kolmogorov complexity, Solomonoff priors, and Solomonoff kernels as a foundation for kernel-based learning theory; the length-conditioned Solomonoff-type prior $M_{n}$ of Section 2 is the same algorithmic-information substrate. The present diagnostic for spurious time-series correlations applies that substrate in a complementary direction—to the detection of low-complexity coincidence rather than to learning-theoretic generalisation—and its results are consistent with the programme’s central theme that compressibility governs what can be reliably inferred from data.

## Figures and Tables

**Table 1:** The three pairwise regimes in the algorithmic trilemma. Any two of independence, high correlation, and high complexity may hold together, but all three cannot hold simultaneously except by algorithmically non-independent alignment.

Case	Meaning
*Independent + complex *	They should look like unrelated noise, so high correlation is unlikely.
*Independent + highly correlated*	Then they must be simple enough to align by chance.
*Highly correlated + complex*	Then they probably share information; they are not algorithmically independent.

**Table 5:** Interpretation of Pearson correlation together with the joint LZ-complexity screening indicator $J_{\mathrm{LZ}}$. High correlation with high $J_{\mathrm{LZ}}$ is treated as worth further investigation, whereas high correlation with low $J_{\mathrm{LZ}}$ is treated as suspicious because it may be driven by shared low-complexity structure.

Correlation	Complexity $J_{\mathrm{LZ}}$	Interpretation
Low correlation	High $J_{\mathrm{LZ}}$	Complex but apparently unrelated.
High correlation	High $J_{\mathrm{LZ}}$	Worth investigating further.
High correlation	Low $J_{\mathrm{LZ}}$	Likely explained by simple shared structure.
Low correlation	Low $J_{\mathrm{LZ}}$	Simple but not correlated.

**Table 6:** Existing spurious-correlation diagnostics and the proposed $J_{\mathrm{LZ}}$ screen. Each method targets a different aspect of the problem and is best understood as complementary rather than competing.

Method	Targets	Limitation
Surrogate tests (random-shuffle, phase-randomised, AAFT)	Departure from a chosen i.i.d. or linear-Gaussian null	The null may be inappropriate for simple monotone trends; both series can pass the null and still be “trivially” related
Cointegration/unit-root tests (ADF, Johansen)	Non-stationary stochastic correlations (Yule phenomenon)	Does not detect simple deterministic-trend spuriousness
MDL causal discovery (GLOBE, CASCADE)	Causal structure/model selection on a graph	Heavier than a single-pair diagnostic; aims at DAG recovery
Normalised Compression Distance (NCD)	Algorithmic dependence between two specific series	$O(K^{2})$ pairwise compressions; not designed as a Pearson-correlation reliability filter
$J_{\mathrm{LZ}}$ screen (this paper)	Low-complexity shared structure in correlated pairs	Depends on serialisation/quantisation; not a causal test; requires stationarity pre-step

## Data Availability

No new data were created or analyzed in this study. Data sharing is not applicable to this article.

## Abbreviations

Symbol	**Meaning**
*N*	Length of real-valued time series
*n*	Length of binary sequence (n≈32N under pickle+float32 serialisation)
*M*	Byte-string length after serialisation (M≈4N for pickle+float32, M=8N for float64, M=2N for int16)
$x,y\in R^{N}$	Real-valued time series
$u,v\in {\left\{0,1\right\}}^{n}$	Binary strings (used exclusively in theoretical sections)
K(x)	Prefix Kolmogorov complexity
$K^{A}\left(x\right)$	Kolmogorov complexity relative to oracle *A*
κ(x)	Complexity rate ${\lim\inf}_{r}K\left(x↾r\right)/r$
$\tilde{K}\left(u\right)=K\left(u\right)/n$	Normalised Kolmogorov complexity of a length-*n* binary string *u*
$C_{\mathrm{LZ}}\left(x\right),\;{\tilde{C}}_{\mathrm{LZ}}\left(x\right)$	LZ-compressed length (bytes) and its ratio to *M*
$H_{F}\left(x\right),\;\tilde{H}\left(x\right)$	Spectral entropy and its normalised version
$J_{\mathrm{LZ}}\left(x,y\right)$	Joint complexity screening indicator (primary): $\min\{{\tilde{C}}_{\mathrm{LZ}}\left(x\right),{\tilde{C}}_{\mathrm{LZ}}\left(y\right)\}$
$J_{\mathrm{geom}}\left(x,y\right)$	Geometric-mean alternative (operationally equivalent): $\sqrt{{\tilde{C}}_{\mathrm{LZ}}\left(x\right){\tilde{C}}_{\mathrm{LZ}}\left(y\right)}$
$S_{\mathrm{LZ}}\left(x,y\right)$	Complexity-similarity indicator (diagnostic only): $-|{\tilde{C}}_{\mathrm{LZ}}\left(x\right)-{\tilde{C}}_{\mathrm{LZ}}\left(y\right)|$
ρ(x,y)	Empirical Pearson correlation
$\hat{\rho }\left(u,v\right)=1-2d_{H}\left(u,v\right)/n$	Bipolar overlap (Hamming correlation)
$\tau ,\;\delta_{\tau },\;h\left(\cdot \right)$	Correlation threshold, radius $\delta_{\tau }=\left(1-\tau \right)/2$, binary entropy function
α,β	Complexity thresholds (normalised)
$\varepsilon ,\;\varepsilon_{c}$	Coupling strength and synchronisation threshold
$\lambda_{i},\;\lambda_{T},\;\lambda_{\max}$	Lyapunov exponents, transversal and maximal
$h_{\mathrm{KS}},\;D_{\mathrm{KY}}$	Kolmogorov–Sinai entropy and Kaplan–Yorke dimension
$H_{i},\;\dim_{H}$	Hurst parameter, Hausdorff dimension (mfBm)
$M_{n}$	Length-conditioned discrete Solomonoff prior
$Z_{n}=\sum_{z\in {\left\{0,1\right\}}^{n}}2^{-K(z)}$	Normalisation constant of $M_{n}$
θ	Threshold on $J_{\mathrm{LZ}}$ for pair acceptance

## Appendix A. Pearson Volume Bound

While the main text uses the Hamming correlation for geometric clarity, the same $h(\delta_{\tau })$ capacity ceiling applies to the standard empirical Pearson correlation, introducing only an additional polynomial factor ${\left(n+1\right)}^{2}$.

**Definition A1** 
(Empirical Pearson correlation)**.** *For binary sequences $x,y\in {\left\{0,1\right\}}^{n}$, the empirical Pearson correlation ${\hat{\rho }}_{\mathrm{Pearson}}\left(x,y\right)$ is the standard statistical correlation coefficient computed by treating their bits as real numbers:*

$${\hat{\rho }}_{\mathrm{Pearson}}\left(x,y\right)\;=\;\frac{\sum_{i=1}^{n}\left(x_{i}-\mu_{x}\right)\left(y_{i}-\mu_{y}\right)}{n\sigma_{x}\sigma_{y}}$$

*where $\mu_{x},\mu_{y}$ are the empirical means and $\sigma_{x},\sigma_{y}$ are the empirical standard deviations.*

**Lemma A1** 
(Pearson Volume Bound)**.** *Let $x\in {\left\{0,1\right\}}^{n}$ be a fixed binary sequence with non-zero empirical variance (the Pearson correlation is otherwise undefined) and τ∈(0,1) a correlation threshold. Let $S_{\tau }\left(x\right)=\left\{y\in {\left\{0,1\right\}}^{n}:\sigma_{y}>0,\;{\hat{\rho }}_{\mathrm{Pearson}}\left(x,y\right)\geq \tau \right\}$ be the set of valid correlated sequences (we count only y with non-zero empirical variance). The cardinality of this set is bounded by*

$$|S_{\tau }{\left(x\right)|\;\leq \;\left(n+1\right)}^{2}\;2^{nh(\delta_{\tau })}$$

*where $\delta_{\tau }=\frac{1-\tau }{2}$ and h(p) is the binary entropy function.*

**Proof.** The sequence pair (x,y) defines an empirical joint probability distribution $P_{X,Y}\left(a,b\right)=\left|\left\{1\leq t\leq n:\left(x_{t},y_{t}\right)=\left(a,b\right)\right\}\right|/n$ for $\left(a,b\right)\in {\left\{0,1\right\}}^{2}$. The empirical Pearson correlation ${\hat{\rho }}_{\mathrm{Pearson}}\left(x,y\right)$ is exactly the correlation coefficient ρ of this joint distribution. For a fixed sequence *x*, its marginal empirical type $P_{X}$ (the proportion of 0s and 1s in *x*) is fixed. By the Method of Types ([72], Section 11.1), the number of sequences *y* that realise a specific joint type $P_{X,Y}$ alongside *x* is bounded by $2^{nH(Y|X)}$, where H(Y|X) is the conditional Shannon entropy of the empirical distribution. Given the fixed marginal $P_{X}$, there are at most $\left(n_{0}+1\right)\left(n_{1}+1\right)\leq {\left(n+1\right)}^{2}$ joint empirical types compatible with $P_{X}$ (where $n_{0},n_{1}$ are the marginal counts), so the total number of valid correlated sequences is bounded by:

$$|S_{\tau }{\left(x\right)|\;\leq \;\left(n+1\right)}^{2}\max_{P_{X,Y}:\rho \geq \tau }2^{nH(Y|X)}$$

To bound this maximum entropy, let V=P(Y=1|X) be a random variable taking the value P(Y=1|X=0) with probability P(X=0) and P(Y=1|X=1) with probability P(X=1). The conditional entropy is H(Y|X)=E[h(V)]. By the law of total variance, $\sigma_{Y}^{2}=E\left[\mathrm{Var}\left(Y|X\right)\right]+\mathrm{Var}\left(E\left[Y|X\right]\right)$. Since *Y* is binary, its conditional variance is exactly Var(Y|X)=V(1−V). Because *X* is also binary, any function of *X* is perfectly linear, meaning the conditional expectation is identically the linear regression: $E\left[Y|X\right]=\mu_{Y}+\rho \frac{\sigma_{Y}}{\sigma_{X}}\left(X-\mu_{X}\right)$. Taking the variance yields $\mathrm{Var}\left(E\left[Y|X\right]\right)=\rho^{2}\sigma_{Y}^{2}$. Substituting these into the law of total variance yields:

$$E\left[\;V\left(1-V\right)\;\right]\;=\;\sigma_{Y}^{2}-\rho^{2}\sigma_{Y}^{2}\;=\;\sigma_{Y}^{2}\left(1-\rho^{2}\right)$$

Define the function $f\left(v\right)=h\left(\frac{1}{2}-\frac{1}{2}\sqrt{1-4v}\right)$ for v∈[0,1/4] so that h(V)=f(V(1−V)). One can verify by differentiation that f(v) is strictly concave and monotone increasing on its domain. Therefore, by Jensen’s inequality applied to the concave function *f*:

$$H\left(Y|X\right)\;=\;E\left[f\left(V\left(1-V\right)\right)\right]\;\leq \;f\left(E\left[V\left(1-V\right)\right]\right)\;=\;f\left(\sigma_{Y}^{2}\left(1-\rho^{2}\right)\right)$$

Because *f* is monotone increasing and the marginal variance of any binary variable is bounded by $\sigma_{Y}^{2}\leq 1/4$, the conditional entropy reaches its absolute global maximum when $\sigma_{Y}^{2}=1/4$:

$$H\left(Y|X\right)\;\leq \;f\left(\frac{1-\rho^{2}}{4}\right)\;=\;h\left(\frac{1}{2}-\frac{1}{2}\sqrt{1-4\left(\frac{1-\rho^{2}}{4}\right)}\right)\;=\;h\left(\frac{1-|\rho |}{2}\right)$$

Because our threshold requires ρ≥τ>0, the entropy is upper-bounded by $h\left(\frac{1-\tau }{2}\right)=h\left(\delta_{\tau }\right)$. Substituting this uniform limit into the Method of Types bound completes the proof.    □

## Appendix B. Multivariate Fractional Brownian Motion: Model Details

**Proposition A1** 
(Cross-covariance of mfBm, [45])**.** *A bivariate mfBm $\left(X_{1},X_{2}\right)$ with Hurst parameters $\left(H_{1},H_{2}\right)$ has cross-covariance for $H_{1}+H_{2}\neq 1$:*

$$E\left[X_{i}\left(s\right)X_{j}\left(t\right)\right]=\frac{\sigma_{i}\sigma_{j}}{2}\left\{\left(\rho_{ij}+\eta_{ij}\;\mathrm{sgn}\left(s\right)\right){\left|s\right|}^{H_{i}+H_{j}}+\left(\rho_{ij}-\eta_{ij}\;\mathrm{sgn}\left(t\right)\right){\left|t\right|}^{H_{i}+H_{j}}-\left(\rho_{ij}-\eta_{ij}\;\mathrm{sgn}\left(t-s\right)\right){\left|t-s\right|}^{H_{i}+H_{j}}\right\},$$

*where $\rho_{ij}=\mathrm{corr}\left(X_{i}\left(1\right),X_{j}\left(1\right)\right)$ and $\eta_{ij}=-\eta_{ji}$ is the asymmetry parameter.*

### Simulation

The mfBm simulations in this paper use the standard circulant-embedding construction described in the cited references [45,73]. Concretely, the Wood–Chan algorithm [47] constructs exact fGn samples by embedding the covariance matrix determined by Proposition A1 in a circulant matrix and using the FFT to generate samples with the exact covariance structure; the bivariate paths are then obtained by cumulative summation of the coupled fGn increments. Since the explicit spectral factorisation of the cross-spectral density is not used in any of the theoretical arguments of this paper, we omit the full derivation and provide only the model definition above together with the simulation parameters needed for reproducibility (Appendix D); the code repository contains the implementation used for the figures.

## Appendix C. Kolmogorov Complexity via the Discrete Fourier Transform

The Fourier basis offers one constrained description language, and the sparsest exact description in that language provides a useful operational complexity measure: the smaller the exact lossless Fourier representation, the lower the estimated complexity. This is because spatiotemporal patterns with stronger regularity can be reconstructed from fewer retained coefficients, while more irregular or chaotic patterns need denser frequency support.

Ordinarily, Fourier compression is lossy because frequency filtering removes information; however, [44] showed that for *binary* images, some loss can be corrected during a final binarisation step. This means that if the inverse-transformed image is only perturbed mildly (i.e., by removing insignificant Fourier coefficients), thresholding is able to resolve the loss in the final reconstruction to regain the original binary image.

Let $s\in {\left\{0,1\right\}}^{W\times H}$ be a binary image, F(s) its 2D discrete Fourier transform, and $m\in {\left\{0,1\right\}}^{W\times H}$ a binary mask that selects which Fourier coefficients to preserve or drop. The compressed representation is thus z=m⊙F(s), where ⊙ denotes element-wise multiplication. The key insight is that $\hat{s}$ can equal *s* despite *z* being a filtered version of F(s) (retaining the minimal subset of most significant coefficients). Reconstruction proceeds by $\hat{s}=1_{>\vartheta }\;\left(F^{-1}\left(z\right)\right)$, where $1_{>\vartheta }$ is a hard thresholding operator with threshold ϑ (denoted ϑ rather than θ to avoid conflict with the $J_{\mathrm{LZ}}$ screening threshold θ of Section 7). So the final image $\hat{s}$ becomes *lossless after quantisation* (even though the Fourier filtering step by itself is lossy).

Although designed for 2D binary images, the same principles naturally extend to 1D sequences, $s\in {\left\{0,1\right\}}^{N}$, by replacing the 2D DFT with its 1D counterpart. A sparse subset of Fourier coefficients sufficient to reconstruct the original sequence exactly after inverse transformation followed by thresholding is then found the same as before. The complexity of *s* can be quantified by the minimal support of the filter mask $m\in {\left\{0,1\right\}}^{N}$ that selects the optimal subset of frequency components.

This perspective provides a concrete proxy for Kolmogorov complexity under a restricted description language (i.e., the complexity of *s* is approximated by the size of the smallest Fourier support that yields exact recovery under thresholding). This basis-dependent measure offers a practically tractable way to relate spectral sparsity to structural regularity.

## Appendix D. Reproducibility

We collect here the practical settings used to generate the figures of Section 3, Section 4, Section 5, Section 6, Section 7 and Section 8. All experiments were run in Python 3.11 on a single CPU core (Intel i7-class workstation), using the following packages: numpy 1.26, scipy 1.11, matplotlib 3.8, zlib (Python standard library, DEFLATE level 6), pickle protocol 4. fGn/fBm sampling uses the Wood–Chan circulant-embedding method [47] as implemented using NumPy primitives.

### Appendix D.1. Logistic-Map Experiments (Section 4)

Initial conditions $x_{0}=0.1$, $y_{0}=0.2$; transient of 1000 iterates discarded; record length N=5000. The coupling sweep uses 51 equally spaced ε values in [0,0.5]. The false-positive rate experiment uses 200 independent pairs at each of r∈[2.8,3.99] (sampled on a uniform grid of width 0.01), with independent random initial conditions drawn uniformly in (0.05,0.95) for each pair. The synchronisation criterion is $|x_{n}-y_{n}|<10^{-6}$ sustained for the final 500 iterates. The threshold |ρ|>0.3 used in Figure 5a is the value at which the periodic regime (r=3.4) trivially exceeds the threshold (since ρ∈{−1,+1} exactly there), and the chaotic regime (r=3.9) sits well below it.

### Appendix D.2. mfBm Experiments (Section 5)

Path length N=2000; 500 independent realisations per Hurst value; Hurst grid H∈{0.05,0.10,0.20,0.30,0.40,0.50,0.60,0.70,0.75,0.80,0.85,0.90}. Wood–Chan circulant embedding requires positive-semidefinite extension and fails at H≥0.95 for N=2000, so the grid is truncated at H=0.90. The false-positive threshold |ρ|>0.1 is chosen because the effect at stationary Gaussian processes is subtler than the deterministic logistic regime; the monotone *H*-dependence of the false-positive rate persists for thresholds 0.05–0.20 (only the absolute level shifts).

### Appendix D.3. Serialisation Comparison (Table 2)

Logistic series at N=5000, 20 realisations per row; fGn/fBm series at N=2000, 30 realisations per row. Standard deviations are below 0.02 in every reported cell. Quantisation for int16 and int8 uses uniform binning across the observed dynamic range of each individual realisation (no global scaling).

### Appendix D.4. NCD Comparison (Figure 8)

NCD is evaluated via three zlib compressions per pair: C(x), C(y), C(xy), where xy denotes the concatenation of the two pickle+float32 byte representations with no inter-stream marker. zlib compression level 6 (the default) is used throughout.

### Appendix D.5. Threshold and fGn Family Calibration

The fGn false-positive curve of Figure 6b locates the 5% transition at H≈0.83. This transition has no absolute ${\tilde{C}}_{\mathrm{LZ}}$ coordinate: fGn increments saturate near ${\tilde{C}}_{\mathrm{LZ}}\approx 0.93$ under pickle+float32 and remain near ${\tilde{C}}_{\mathrm{LZ}}\approx 0.945$ under int8 for all *H* (the int8 drop to ≈0.5 belongs to the non-stationary fBm paths, not the increments; Table 2). The threshold therefore cannot be applied as a fixed or rank-based $J_{\mathrm{LZ}}$ cutoff for fGn increments; the false-positive risk must be read from an *H*- or memory-parameter calibration curve. The value θ=0.3 is the operating point used for the bounded logistic pipeline and for data classes/encodings where $J_{\mathrm{LZ}}$ has calibrated dynamic range.

### Appendix D.6. Source Code

The reproduction package accompanying this revision contains the scripts used to generate the figures and tables, including the logistic-map sweeps, fGn/fBm simulations, serialisation comparison, NCD comparison, and threshold-calibration tables. A permanent public repository link will be provided in the published version.
